# Whole-genome resequencing provides insights into the evolution and divergence of the native domestic yaks of the Qinghai–Tibet Plateau

**DOI:** 10.1186/s12862-020-01702-8

**Published:** 2020-10-27

**Authors:** Zhi-xin Chai, Jin-wei Xin, Cheng-fu Zhang, Dawayangla, Luosang, Qiang Zhang, Pingcuozhandui, Chao Li, Yong Zhu, Han-wen Cao, Hui Wang, Jian-lin Han, Qiu-mei Ji, Jin-cheng Zhong

**Affiliations:** 1 Key Laboratory of Qinghai-Tibetan Plateau Animal Genetic Resource Reservation and Utilization, Sichuan Province and Ministry of Education, Southwest Minzu University, Chengdu, China; 2 State Key Laboratory of Hulless Barley and Yak Germplasm Resources and Genetic Improvement, Institute of Animal Science and Veterinary Research, Tibet Academy of Agricultural and Animal Husbandry Sciences, Lhasa, China; 3 CAAS-ILRI Joint Laboratory on Livestock and Forage Genetic Resources, Institute of Animal Science, Chinese Academy of Agriculture Sciences (CAAS), Beijing, China

**Keywords:** Domestication, Plateau adaptability, Gene exchange, Bovidae, Hybrids, Selective

## Abstract

**Background:**

On the Qinghai–Tibet Plateau, known as the roof ridge of the world, the yak is a precious cattle species that has been indispensable to the human beings living in this high-altitude area. However, the origin of domestication, dispersal route, and the divergence of domestic yaks from different areas are poorly understood.

**Results:**

Here, we resequenced the genome of 91 domestic yak individuals from 31 populations and 1 wild yaks throughout China. Using a population genomics approach, we observed considerable genetic variation. Phylogenetic analysis suggested that the earliest domestications of yak occurred in the south-eastern QTP, followed by dispersal to the west QTP and northeast to SiChuang, Gansu, and Qinghai by two routes. Interestingly, we also found potential associations between the distribution of some breeds and historical trade routes such as the Silk Road and Tang-Tibet Ancient Road. Selective analysis identified 11 genes showing differentiation between domesticated and wild yaks and the potentially positively selected genes in each group were identified and compared among domesticated groups. We also detected an unbalanced pattern of introgression among domestic yak, wild yak, and Tibetan cattle.

**Conclusions:**

Our research revealed population genetic evidence for three groups of domestic yaks. In addition to providing genomic evidence for the domestication history of yaks, we identified potential selected genes and introgression, which provide a theoretical basis and resources for the selective breeding of superior characters and high-quality yak.

## Background

The domestication of animals provided a stable source of food, labor, and hides, which played an important role in the lifestyle changes of humans from hunter gatherer to agricultural settlement [1]. On the Qinghai–Tibet Plateau (QTP), known as the roof ridge of the world, the average altitude is over 4000 m, where most plants and animals cannot survive because of the harsh climate, hypoxia, and low atmospheric pressure [2, 3]. However, the Tibetan people came to this land and created a splendid civilization with the most indispensable assistance of their domesticated yaks. Unlike other large herbivorous livestock (average weight greater than 40 kg), Tibetan sheep, which spread to QTP after the domestication [4, 5], the yak is an endemic species and domestication of wild yak occurred on the QTP [6]. Thus, the origin of domestication of yaks and their dispersal route is an important strand of evidence for the history of human migration, exploitation, and development on the QTP. In addition, the detection of genomic differences among domestic yaks may help elucidate the underlying mechanisms of adaptation and facilitate selective breeding.

Previous studies have investigated yaks at archaeological [7–9], mitochondrial [10–12], whole genome landscape [13] and population resequencing [14] levels. Whole genome sequencing and comparative genomics analysis in yak identified the expansion of gene families related to sensory perception and energy metabolism and some positively selected genes related to hypoxia and nutrition metabolism. Population genetic analysis identified 209 genes which relate to behavior and tameness and suggest that the domestication of yaks occurred in the QTP ~ 7300 years BP, followed by a six-fold increase in yak population size by 3600 years BP. Most previous studies focused on the differentiation and difference between domestic yaks and wild yaks. But the location of their earliest domestication, dispersal direction, and the difference among domestic yaks from different areas have not previously been studied at the genomic level.

Compared with other livestock, domestic yaks have a lower degree of domestication. Domestic yaks have a wide range of interactions with wild yaks and genetic exchange occurs and is hard to avoid. In addition, genetic exchange among domestic yaks from different areas occurs frequently because of human activities. The phenotypic and character differences of domestic yaks are not conspicuous. To examine intraspecific genetic diversification and geographical distributions of genetic lineages, we performed whole genome resequencing of 91 domestic yaks distributed throughout the QTP, from XiZang, QingHai, SiChuang, YunNan, GanSu, and XinJiang. Analyses of population genetics, selection, and demographic history built a database of genetic diversification resources for domestic yaks and revealed a series of interesting discoveries regarding their domestication and dispersal.

## Results

### Genome resequencing and genetic variation

We generated whole-genome sequences of 91 domestic yaks from 31 different locations and 1 wild yak from the QTP (Additional file 1: Fig. S1). Sequence data from a further 17 wild yaks were downloaded from NCBI for analysis (Additional file 1: Table S1). The samples of domestic yak were widely distributed and include most of the nationally recognized varieties. In total, ~ 21 billion raw reads and ~ 2078 Gb of aligned high-quality data with an average depth of 7.2× were generated using Illumina sequencing technology (Additional file 1: Table S2). After SNP calling and subsequent stringent quality control, we obtained 44,296,018 high-quality SNPs for all 108 individuals, with a range of 6,019,569 to 14,518,382 SNPs per individual. Most of the SNPs were located in intergenic regions: 144,673 in exonic regions and 27,947 nonsynonymous SNPs. The SNP distribution characteristics were similar to those of other livestock like pigs [15] sheep [16].

The mutations or genotypes specific to the wild yak genomes will provide an important resource for breeding. We identified 330,962 wild group-specific SNPs; 2733 of these are located in coding regions and involve 1009 genes that were enriched for olfactory-related functions. The living environment of wild yaks is worse than domestic yaks. Wild yaks living on the higher altitude and don’t have stable pastures, they need not only to prowl for food, but also to avoid predators (wolf). The unusual olfactory might helped them to adapt to their environment and avoid predators. We also identified variations in gene regions specific to each group of domestic yaks. Although the differences among groups were not obvious, the group-specific variation at the genomic level was significative and will help to provide an insight into the relevant unique traits. In our samples, the most obvious phenotypic character divergence is the pure white hair specific to the Tianzhu group [17]. We identified 10 specific SNPs shared in all three TZ individuals, but no nonsynonymous SNPs were found.

### Population genetic phylogeny

**Fig. 1:**
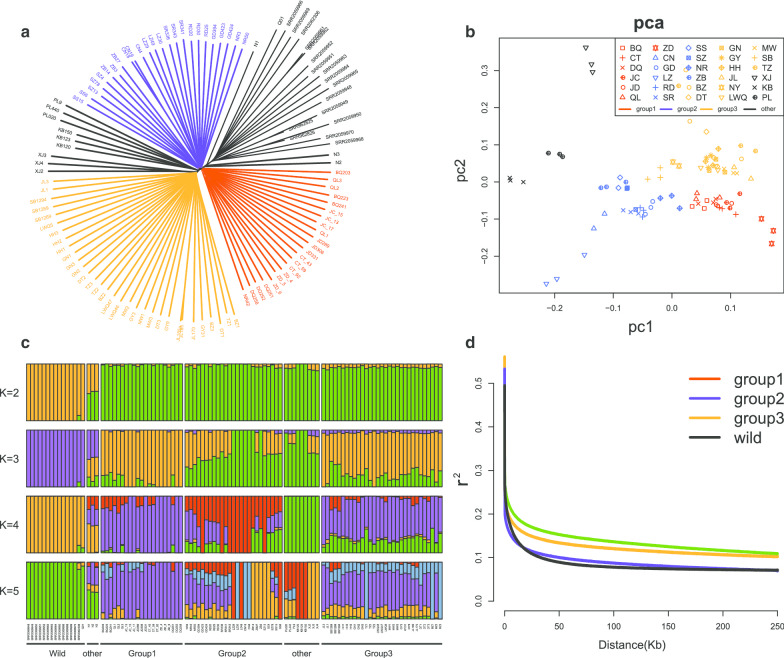
Population genetic structure of the yak populations studied. **a** Neighbor-joining (NJ) tree of 109 yak individuals generated using TreeBest software, with wild yaks as the outgroup. The group 1, group 2 and group 3 of domestic yaks are colored with red, blue and orange. **b** Plots of principal components 1 and 2 from PCA analysis of 92 Chinese domestic yak individuals using the GCTA software. The colors of samples are same with the **a**. **c** Population genetic structure of domestic yak and wild yak inferred from the ADMIXTURE analyses (K = 2–5) using whole-genome SNPs. **d** Linkage disequilibrium (LD) decay for the four separate groups/subgroups of populations measured by r^2^

To identify the genetic relationships between domestic yaks, we constructed a phylogenetic tree of yak SNPs using the neighbor-joining method with wild yaks as an outgroup (Fig. 1). The results showed a relatively close genetic distance and indicated that the domestic yaks were divided into three main branches.

The branch containing BQ, JC, JD, CT, ZD, and DQ separated first from wild yak. These areas are on the southeastern edge of the QTP, and, except for ZD, they are located at very similar latitudes. The Changdu area (N31°, E97°), at the center of these regions, was historically the populated area of the QTP. In conclusion, we suggest that the Changdu area was most likely the origin of the domesticated yaks and that they first dispersed to the east and west to BaQing and JingChuan, respectively.

The second branch in the phylogenetic tree contained NR, GD, RD, SR, LZ, CN, ZB, SZ, SS, (KB, PL) and showed a small genetic distance from the first branch. These areas represent the core region of Tibet, including the central, west, and south of Tibet, suggesting that following successful domestication, yaks were gradually brought to the hinterland of the QTP. We defined this as the second dispersal of domestic yaks through the east of Tibet to the south and west.

The third branch of phylogenetic tree contained XJ, JL, SB, LWQ, HH, GN, TZ, MW, DT, NY, GY, and BZ. Most of these are located at the edge of QTP in Qinghai, Gansu, Sichuang, Shanxi, Yunnan, and XinJiang. This suggested that the third dispersal of domestic yaks was from the center to the periphery of the plateau. Moreover, we found that the diversity of the third branch was higher than that of the other two branches. This is not only related to the hybridization of yaks in trade, but also to interspecies hybridization with wild yaks or cattle [18, 19], for example the DaTong yak is a new breed created by hybridization with wild yaks. In comparison, most breeds in the second branch showed a lower diversity that was consistent with a more closed trading environment, harsher living environment, and smaller population (Additional file 1: Table S3). In addition, we constructed a phylogenetic tree including all the domestic yak data of Qiu et al. [14]. The topology was similar for the samples from the present study, but the downloaded data formed narrow disordered clusters in the second branch and the third branch. This may reflect our more extensive sampling and more accurately defined breeds in the present study (Additional file 1: Fig. S2).

### Population component, diversity, and linkage disequilibrium

Principal component analysis (PCA) and Bayesian model-based clustering analysis were employed to examine the phylogenetic groupings and provide any additional evidence. The PCA did not show significant separation among the samples of domestic yak, consistent with a single origin of domestication and the close genetic distance shown in the phylogenetic tree (Additional file 1: Fig. S3). However, when the wild yaks and ambiguous breeds (KB, PL, XJ) were excluded from the analysis, the three separate groupings of domestic yaks were evident (Fig. 1). Furthermore, the first principal component (PC1) separated group 1 and group 2 from group 3; PC2 separated group 1 and group 3 from group 2; and PC3 seemed to separate group 1 from group 2 and group 3, but this was not clear (Additional file 1: Fig. S4). In the clustering analysis performed using ADMIXTURE, with K = 2, the yaks were genetically divided into wild and domestic samples. As K increased (K = 3–5), the domestic samples were not separated into distinct breeds, suggesting extensive genetic admixture among living domesticated yaks (Fig. 1). The genome-wide average θ_Π_ value for domestic groups was 0.93–1.2 × 10^–3^ was similar to that for the wild yaks (1.1 × 10^–3^), which is consistent with previous research [14] (Additional file 1: Table S3). In addition, the higher θ_Π_ of group 3 and the lower θ_Π_ of group 2 support the inferences related to trade and geographical enclosure. Linkage disequilibrium analysis suggested that the wild group exhibited a rapid decay rate and a low level of LD, whereas the group 3 yaks showed an overall slow decay rate and a high level of LD (Fig. 1).

### Selective analysis and comparison

We calculated pairwise FST to quantify the genetic differentiation among the four groups. Pairwise FST ranged from 0.019 to 0.068 with an average of 0.043, which is smaller than that between diverged taurine cattle breeds [20], and is consistent with the gene flow occurring between wild and domestic yaks. Moreover, the FST between groups of domestic yaks ranged from 0.019 (group 2 vs group 3) to 0.027 (group 1 vs group 2), consistent with a very low degree of differentiation among the domestic breeds. FST between domestic and wild yaks ranged from 0.058 to 0.068, with the lowest FST occurring between group 3 and the wild group. This indicates a higher degree of crossbreeding between group 3 breeds and wild yaks, creating hybrid breeds such as the DaTong yak. Group 3 had a lower FST compared to those of the other domestic groups (Additional file 1: Table S4). This further indicated the higher degree of hybridization of group 3 and is consistent with the ADMIXTURE analysis results.

**Fig. 2:**
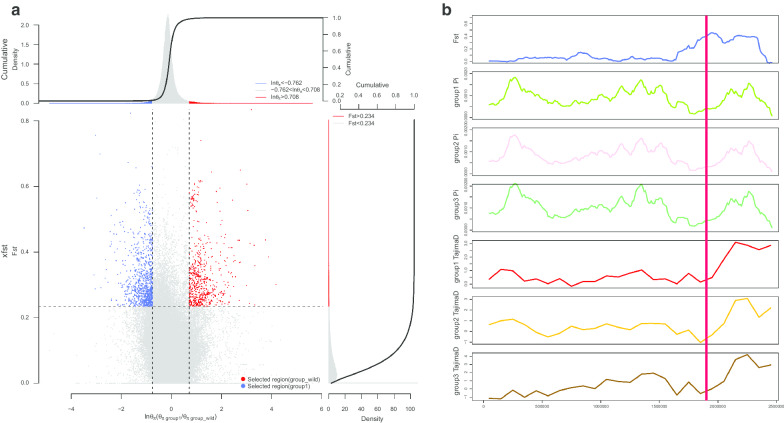
The selective-sweep region and region with extremely high FST values in yaks. **a** Distribution of pi ratio (group 1/wild) and FST of 100 kb windows with 10 kb steps. Red dots represent windows fulfilling the selected regions in wild yaks and blue dots represent windows fulfilling the selected regions in domestic yaks. **b** An example of the extremely high FST (top 0.1%) values region with selection signals in yaks. FST, pi, and Tajima’s D values are plotted using a 100 kb sliding window. The red line indicates the position of the selected gene Bmu002268.1

Regions under directional selection should show specific signatures of variation, including high population differentiation, lower levels of nucleotide diversity, and long-range haplotype homozygosity [21]. To determine whether directional selection might have occurred in groups of domestic yaks, we first explored the genomic landscape of the population differentiation to identify candidate genes. Comparing the extremely high FST values (top 0.1%) with wild yaks using a sliding window analysis, we identified 37, 56, and 39 potential positively selected genes in groups 1, 2, and 3, respectively (Additional file 1: Fig. S5, Table S5). There were 11 genes (THEMIS2, XKR8, SMPDL3B, RPA2, TNFSF15, PACSIN2, TCIRG1, NDUFS8, CHKA, ALDH3B1, and SUV420H1) shared by all the three groups (Fig. 2), which represent the core differentiation genes between domesticated and wild yaks. Four of these genes (TCIRG1, NDUFS8, ALDH3B1, CHKA) play an important role in metabolic pathways. TCIRG1 encodes a subunit of a large protein complex known as a vacuolar H+-ATPase (V-ATPase). This protein helps regulate the pH of cells and their surrounding environment, and V-ATPase-dependent organelle acidification is necessary for intracellular processes such as protein sorting, zymogen activation, and receptor-mediated endocytosis [22]. NDUFS8 encodes a subunit of mitochondrial NADH: ubiquinone oxidoreductase, a multimeric enzyme of the respiratory chain responsible for NADH oxidation, ubiquinone reduction, and the ejection of protons from mitochondria [23]. ALDH3B1 encodes an isozyme that may play a major role in the detoxification of aldehydes generated by alcohol metabolism and lipid peroxidation [24]. CHKA has a key role in phospholipid biosynthesis and may contribute to tumor cell growth [25]. RPA2 can activate the ataxia telangiectasia and Rad3-related protein kinases to involved in DNA metabolism such as DNA replication, repair, recombination, telomere maintenance, and the responses to DNA damage [26]. The PACSIN2 gene encodes a member of the protein kinase C and casein kinase substrate in neurons family and is associated with disease and immunity [27]. Significant differentiation between domesticated and wild yaks was identified in 16,17, and 4 genes in groups 1, 2, and 3, respectively, which suggests differences in selection among the different groups of domestic yaks (Additional file 1: Fig. S5).

For a more global comparison of the selection differences among domestic yaks, we relaxed the selection threshold to the top 1% FST region and performed the enrichment analysis of candidate genes. When comparing group 1 with group 2, the top 1% FST region contained 136 genes that were enriched for the GO terms MHC related (GO:0002504, GO:0042613) and molybdenum ion binding (GO:0030151). Several disease pathways were enriched such as type I diabetes mellitus, graft-versus-host disease, rheumatoid arthritis, asthma, Leishmaniasis, and autoimmune thyroid disease (Additional file 1: Table S6, Fig. S6). Some immune related pathways were also enriched, such as allograft rejection, intestinal immune network for IgA production, antigen processing and presentation, and hematopoietic cell lineage. Thus, some of these genes may contribute to adaptation to the extremely similar but locally distinct environments of grass, insects, and climate. The comparison between group 1 and group 3 identified 206 selection difference genes that were enriched for similar GO and KEGG pathways to those mentioned above, but not molybdenum ion binding and hematopoietic cell lineage (Additional file 1: Table S6). The comparison between group 2 and group 3 identified 185 genes that were not significantly enriched for any pathways. A greater number of genes were identified for group 3 than for the other groups, but there was less enrichment, which may be related to their extensive distribution or higher degree of hybridization.

To better understand the selection acting on the three domestic yak groups, we identified the potential selective-sweep region using the signatures of high FST and greater difference value of pi [14, 16] (Additional file 1: Figs. S7–S9). We identified 298, 365, and 383 selective-sweep genes in groups 1, 2, and 3, respectively, and 70 of these were shared by all domestic yak groups, which reflects the influence of domestication (Additional file 1: Fig. S10, Table S7). Some of these genes were related to energy metabolism (PFKFB1, SLC25A10, MRPL12), nerve development and growth (ATP2B2, CACNA1B, GHRL), and phagocytes (ARFGAP3, HGS, CCDC137, ACTG1, ZC3H3, XKR7). PFKFB1 encodes a member of the bifunctional 6-phosphofructo-2-kinase family that forms a homodimer that catalyzes both the synthesis and degradation of fructose-2,6-biphosphate using independent catalytic domains [28]. GHRL is the ligand for growth hormone secretagogue receptor type 1 (GHSR), which induces the release of growth hormone from the pituitary. This has an appetite-stimulating effect, induces adiposity, stimulates gastric acid secretion, and is involved in growth regulation [29, 30]. The selections of metabolic, organ development will be beneficial to their increased reproductive, the selection in nerve development probably have contributed to the taming of yaks, the selection in phagocytes and response to stimulus will be beneficial to their immunity and livability. For the selection genes specific to the three domestic groups, we found the main functions of group 1 genes are immunity and disease; two genes (KDM1A, VEGFD) related to primitive erythrocyte differentiation were found in group 2 [31, 32]; and the specific selection genes of group 3 function in disease and metabolism. In addition, the wild yak’s selective-sweep region may indicate the direction of natural selection. We found 24 overlap genes that were also identified for all three domestic groups and were enriched in functions of pathogenic *Escherichia coli* infection and immunity (leukocyte transendothelial migration, phagosome) (Additional file 1: Table S8).

### Introgression analysis in Yaks

**Fig. 3:**
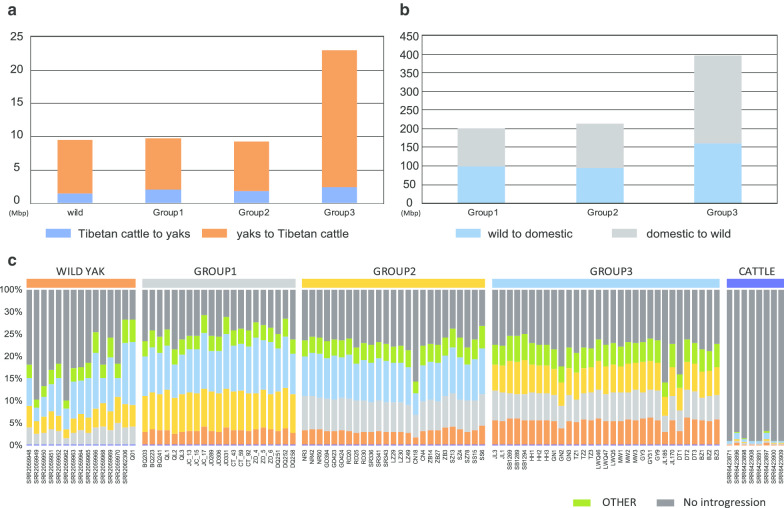
The distribution of introgression among wild yak, Tibetan cattle, and three group of domestic yaks. **a** Comparison of introgression from Tibetan cattle to yaks and vice versa for each group. **b** Comparison of introgression from wild yak to domestic yaks and vice versa for each group. **c** The overall pattern of introgression in each individual. The OTHER group contained the individuals from Xinjiang, Pali, Kangbu, and Geermu, which were difficult to distinguish in PCA

Introgression has occurred extensively in bovine species [18, 19, 33]. We identified gene introgression between domestic yaks, wild yaks, and Tibetan cattle using ChromoPainter [34] software, and found an interesting phenomenon of unbalanced introgression among these groups (Fig. 3, Additional file 2: Table S9). First, the introgression from Tibetan cattle to yaks was far less than that from yaks to Tibetan cattle, for both wild yaks (1.5 M vs 8 M) and domestic yaks (2.1 M vs 44.6 M). Previous studies also reported unbalanced introgression of 5.54 M (from Tibetan cattle to yaks) vs 30.7 M (from yaks to Tibetan cattle) [19]. It is noteworthy that introgression between Tibetan cattle and domestic yaks is more frequent than that between Tibetan cattle and wild yaks. Second, the introgression from wild yaks into domestic yaks was far less than that from domestic yaks into wild yaks (118 M vs 455 M).

To further explore the influence of introgression on yak, we analyzed the related genes in the introgression area. First, we found 521 genes that overlapped the area introgressed from yaks to Tibetan cattle, including 11 genes that function in bile secretion pathways, seven genes associated with endocrine and other factor-regulated calcium reabsorption, nine genes associated with gastric acid secretion, and six glyceride metabolism-related genes (Additional file 1: Table S10). These genes related to digestion and nutrient absorption infiltrated from yaks into Tibetan cattle, helping them better absorb nutrients and energy in the scarce food structure on the plateau. Some introgression genes related to disease and signal transduction also help Tibetan cattle better adapt to the plateau environment. We found 129 genes in the region introgressed from Tibetan cattle into yaks that are involved in signal transduction, physiological regulation (circadian rhythm), metabolism of phosphoinositide pathways, but no significant enrichment (Additional file 1: Table S11).

## Discussion

### The origin of yak domestication

In common, comparing to populations derived through subsequent migration colonization, the populations near to a centre of initial origin are expected to show higher haplotypic and nucleotide diversity as they maintain more ancestral variation. However, this genetic signature might be blurred by the recent gene flow after domestication. Group 3 had a highest θ_Π_ of 0.00111447, compare to 0.000868238 (group 1) and 0.000856952 (group 2), but the widely geography range, disordered phylogeny of three sample from same region and the highest gene exchange among group 3 with Tibetan cattle, wild yaks and other domestic yaks didn’t support it as the origin of domestication. In contrast, the group 1 have an earlier divergence and higher genetic diversity than group 2. The inference of origin is consistent with the history of human transformation from a nomadic society to an agricultural society on the QTP, and is consistent with the previous suggestion through mitochondrial DNA by Guo et al. [35]. In detail, fossil records show that the ancient Qiang nationality lived and formed primitive villages on group 1 area as early as 5000 years ago, and the yaks were domesticated by the Qiang nationality and certainly this great achievement would have improved their lifestyle and helped them exploit, and settle down on, the QTP. Interestingly, the dispersal of domestic yaks in our inference was consistent with the migration of the Qiang nationality. After the dispersal of domestic yaks through the east of Tibet to the south and west, they dispersed from the center to the periphery of the plateau. The wide geographic range of samples in group 3 branch may indicate that this dispersal might have been associated with frequent human activity after the advent of largescale husbandry. Trade might be the principal driver of the dispersal, and given the ancient trading history of China, we suggest that the Tang-Bo Ancient Road and Silk Roads played important roles in the spread of domestic yaks. Although the SB and NY yaks were located closer to the second branch breeds, they clustered in the third branch because they were the tributes collected from other areas owned by the ruling class of Tibet, and many of these tributes were transported through the Tang-Bo Ancient Road. The separate clustering of three individuals of the same breed also reflects the exchange of these yaks.

### The unbalanced introgression

In our result, we found the introgression from Tibetan cattle to yaks was far less than that from yaks to Tibetan cattle. It suggested that the successful adaptation of the cattle to the plateau environment depends on the gene introgression from yak. The introgression between Tibetan cattle and domestic yaks is more frequent than that between Tibetan cattle and wild yaks, we believe that this is related to domestication and breeding in that domestic yaks have more opportunities to exchange genes with Tibetan cattle. We also found the introgression from wild yaks into domestic yaks was far less than that from domestic yaks into wild yaks, this was related to the selective breeding of domestic yaks, however, the small population of wild yaks, the poorer living environment, and the bottleneck effect [36] could also lead to lower retention of introgression in wild yaks. In addition, we detected more introgression between the wild yaks and group 3 than groups 1 and 2, which may be due to the fact that the yaks in group 3 experienced more trade contacts and artificial breeding. For example, the DaTong yak is a new crossbreed between domestic yak and wild yak, and the white hair of Tianzhu yak is also produced by long-term artificial breeding.

In the areas of introgression between domestic yak and wild yak, we found 2608 (domestic to wild) and 307 genes (wild to domestic). These two sets of genes showed similar pathway enrichment results (Additional file 1: Tables S12, S13), for example, focal adhesion, mucin type o-glycan biosynthesis, and arrhythmogenic right ventricular cardiomyopathy (ARVC), indicating that introgression between domestic and wild yaks is likely a balanced exchange in function, which may lead to heterosis.

## Conclusions

Using whole-genome sequencing, our population genomic analyses of domesticated yaks provide new insights into their origin, historical migrations, and introgression events. We have clarified the phylogenetic relationships among the main breeds of domesticated yak, which formed three separate groups. Group 1 was distributed in the southeast of the QTP, which was indicated as the origin of domesticated yaks. Subsequently, yaks spread to the hinterland of Tibet with the ancient Qiang and became group 2. With the increase in human activities, yaks gradually spread to all parts of the plateau, forming group 3 with frequent exchange among breeds. We identified 11 genes related to metabolism and immunity that showed significant selection between domestic yak and wild yak. Although the phenotypic differences among the three groups were subtle, we were able to identify differences among them in genes functioning in disease and energy metabolism. We also characterized the patterns of introgression among domestic yak, wild yak, and Tibetan cattle, and we detected several instances of unbalanced introgression. Higher introgression was detected from yak to Tibetan cattle, and from domestic yak to wild yak, than vice versa. Our study provides an insight into the genetic differences of domestic yak and our results will be beneficial for selective breeding.

## Methods

### Sample preparation, sequencing, and SNP calling

High-quality DNA was extracted from peripheral blood of 92 yaks (91 domesticated yaks and 1 wild yak) sampled in 32 different regions of the Qinghai-Tibet Plateau. Pair-end libraries were constructed with insert sizes of 500 bp and sequenced on an Illumina Hiseq 2000 Sequencer (Illumina, San Diego, CA, USA). In addition, previously published resequencing data from 17 wild yak samples were downloaded from NCBI and used in this analysis [14]. To perform the introgression analysis, we also used sequence data from 8 Tibetan cattle (NCBI accession numbers: SRR6423871, SRR6423896, SRR6423898, SRR6423908, SRR6423891, SRR6423897, SRR6423900, SRR6423909). High-quality reads were aligned against the reference yak genome assembly BosGru_v2.0 using Burrows–Wheeler Aligner software [37]. Bam files were sorted using SortSam and duplicated reads were marked using MarkDuplicates from Picard-Tools 1.56 [38]. The Genome Analysis Toolkit (GATK, version 4.0) [39] was used to perform local realignment of reads to enhance the alignments in the vicinity of indel polymorphisms. Then, the SNPs were detected and filtered using HaplotypeCaller and VariantFiltration commands in GATK. The filter condition of VariantFiltration was "QD < 2.0 || FS > 60.0 || MQ < 40.0 || MQRankSum < − 12.5 || ReadPosRankSum < − 8.0 || SOR > 3.0". Vcftools [40] was used to further filter SNPs with—maf 0.05—max-missing 0.5—min-alleles 2—max-alleles 2.

### Phylogenetic and population genetic analyses

An individual-based Neighbor-Joining phylogenetic tree was constructed from the population scale SNPs using TreeBest [41] software under the p-distances model. We performed PCA with population-scale SNPs using the packages EIGENSOFT [42] and GCTA [43], and the significance level of the eigenvectors was determined using the Tracy–Widom test. Population genetic structure and individual ancestry proportions (admixture) were inferred using the program ADMIXTURE version 1.3.0 [44], which employs a maximum likelihood approach and the expectation–maximization algorithm. We increased the pre-defined genetic clusters from K = 2 to K = 5. Vcftools was used to calculation the population genetic values: pairwise nucleotide variation as a measure of variability (θπ), genetic differentiation (FST), and selection statistics (Tajima’s D, a measure of selection in the genome). A sliding-window approach (100-kb windows sliding in 10-kb steps) was applied to calculation and the per-site basis calculation of pi and FST was performed with -site-pi and -weir-fst-pop. To estimate linkage disequilibrium, we calculated r^2^ between any two loci using PopLDdecay [45]. The average r^2^ value was calculated for pairwise markers in a 500-kb window and averaged across the whole genome. The demographic history of yaks from geographically diverse populations was inferred using a hidden Markov model approach as implemented in pairwise sequentially Markovian coalescent (PSMC) analysis [46] based on SNP distribution.

### Identification of selected regions

For each group, FST values in the top 0.1% region were taken to indicate significant selection and FST values in the top 1% were taken to indicate selected regions. To detect regions with significant signatures of selective sweep, we considered the distribution of the θ π ratios (θ π, population 1/θ π, population 2) and FST values. We used an empirical procedure and selected windows simultaneously with significant low and high θ π ratios (the 2% left and right tails) and significant high FST values (the 2% right tail) of the empirical distribution as regions with strong selective sweep signals along the genome, which should harbor genes that underwent a selective sweep. Protein-coding genes in these outlier windows were treated as candidate positively selected genes. Moreover, EigenGWAS [47] was used to identify the loci under selection, based on a method that uses genome-wide association studies of eigenvectors to identify loci under selection. As EigenGWAS categorizes samples based on PCA, we performed this analysis between each pair of groups of yaks. Subsequently, we determined the genes under selection that were identified by both of these methods. Gene enrichment analysis was performed with EnrichPipelin [48]. Benjamini–Hochberg FDR [49] (false discovery rate) was used to correct the P values for multiple testing. The Gene Ontology categories “Molecular Function,” “Biological Process,” and “Cellular Component,” and the KEGG Pathway were used in these analyses.

### Detection of introgression

For a comprehensive analysis of introgression in yaks, we added data from 8 Tibetan cattle to the population SNP dataset through the same GATK pipeline. The combined VCF dataset was phased using read-aware phasing with SHAPEIT v2.r904 [50–52]. Subsequently, we used Chromopainter in FinSTRUCTUREv2 to identify migrant tracts resulting from introgression among wild yak, domesticated yak, and Tibetan cattle. The program uses a hidden Markov Model to estimate the probability of ancestry at each variable position along the genome using patterns of haplotype similarity. Continuous regions with a probability threshold larger than 0.85 were identified as introgressed tracts.

## Supplementary information

**Additional file 1.** Additional Figures and Tables. **Figures S1–S10**, **Tables S1**–**S8** and **S10**–**S13**: additional data to support the manuscript (see text for references).

**Additional file 2: Table S9.** The introgression statistics of each individual.

## Data Availability

The raw data have been deposited to NCBI. The 92 breeds related data is under bioproject PRJNA670822.

## Abbreviations

QTP: Qinghai–Tibet Plateau
BP: Before present
SNP: Single nucleotide diversity
PCA: Principal component analysis
NJ: Neighbor-joining
LD: Linkage disequilibrium
FST: F-statistics
MHC: Major histocompatibility complex
ARVC: Arrhythmogenic right ventricular cardiomyopathy
