# Supplementary material for: Whole-genome resequencing provides insights into the evolution and divergence of the native domestic yaks of the Qinghai–Tibet Plateau
Source: BMC Evol Biol. 2020 Oct 27;20:137. doi: 10.1186/s12862-020-01702-8 (PMC7590491; doi:10.1186/s12862-020-01702-8)
Supplement: Supplementary file 1 — Additional file 1. Additional Figures and Tables. Figures S1–S10, Tables S1–S8 and S10–S13: additional data to support the manuscript (see text for references). [file 12862_2020_1702_MOESM1_ESM.docx]

**Additional file**


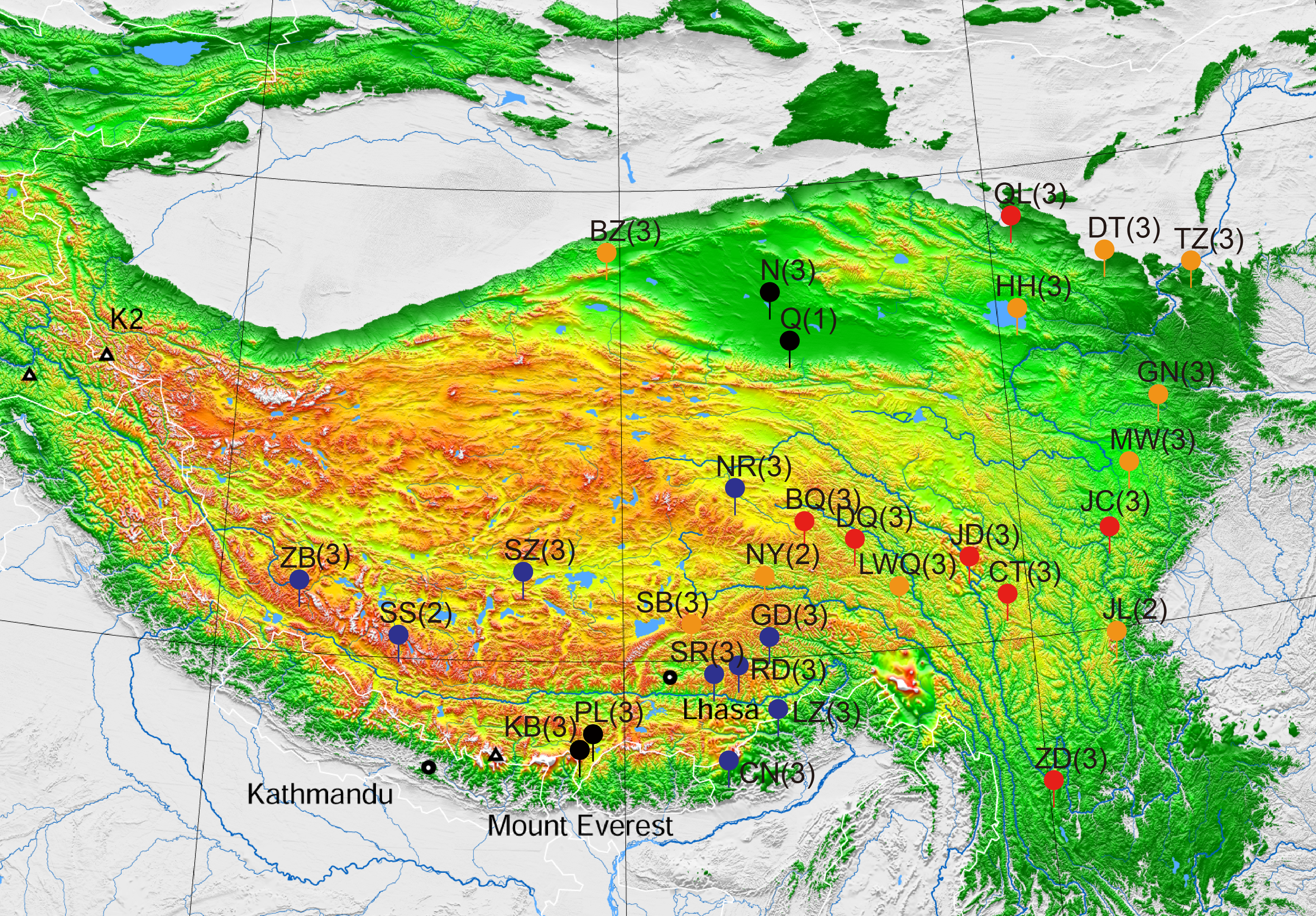


**Figure S1.** **The distribution of yaks of the Qinghai-Tibet Plateau. The group1, group2 and group3 of domestic yak are colored with red, blue and orange. The other samples are colored with black. The numbers in brackets showed the individual of samples in each location**


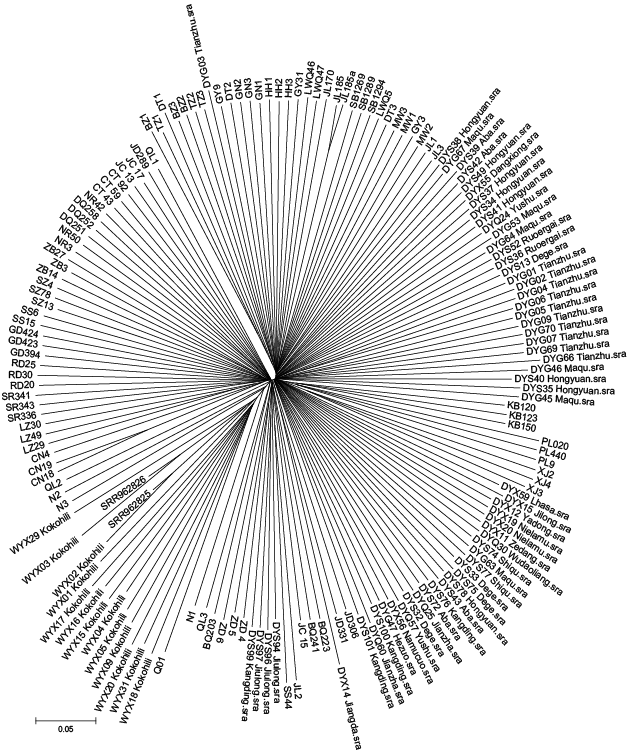


**Figure S2. The phylogenetic tree constructed from the domestic yak data of Qiu et al.**


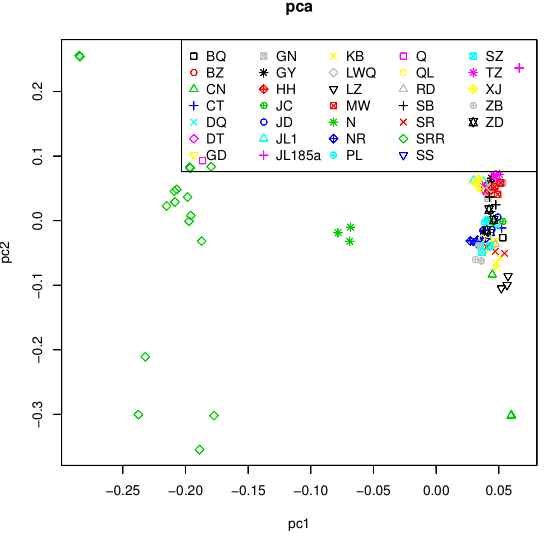


**Figure S3. The PCA of all 109 samples (including wild yaks).**


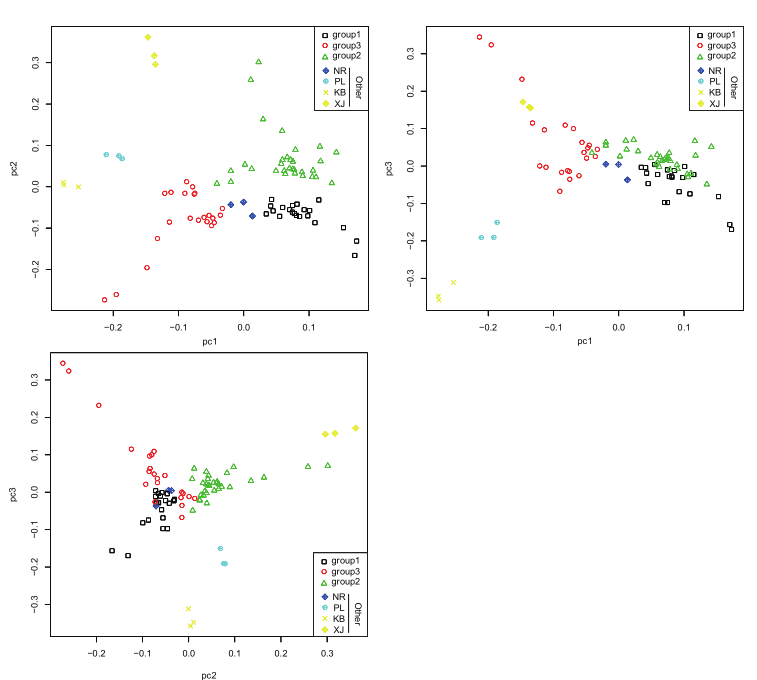


**Figure S4. The PCA of all domestic yak samples**


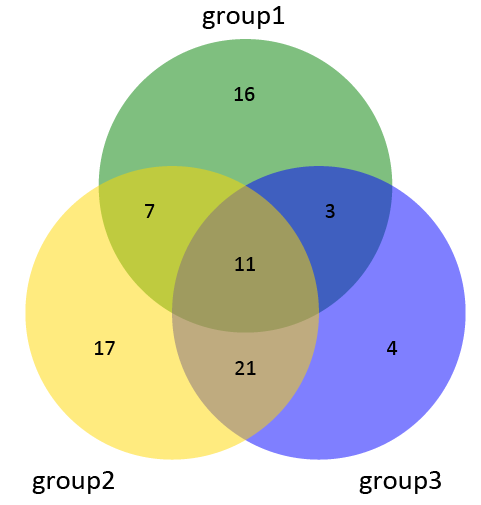


**Figure S5. The Venn diagram of extremely high FST region-related genes**


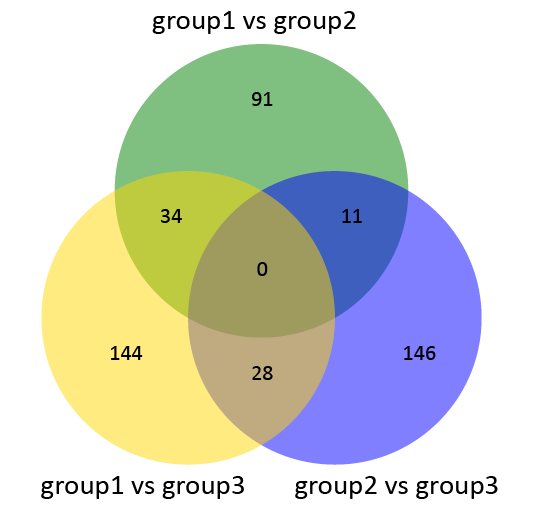


**Figure S6. The Venn diagram of selected genes with FST values in the highest 1% between two groups.**


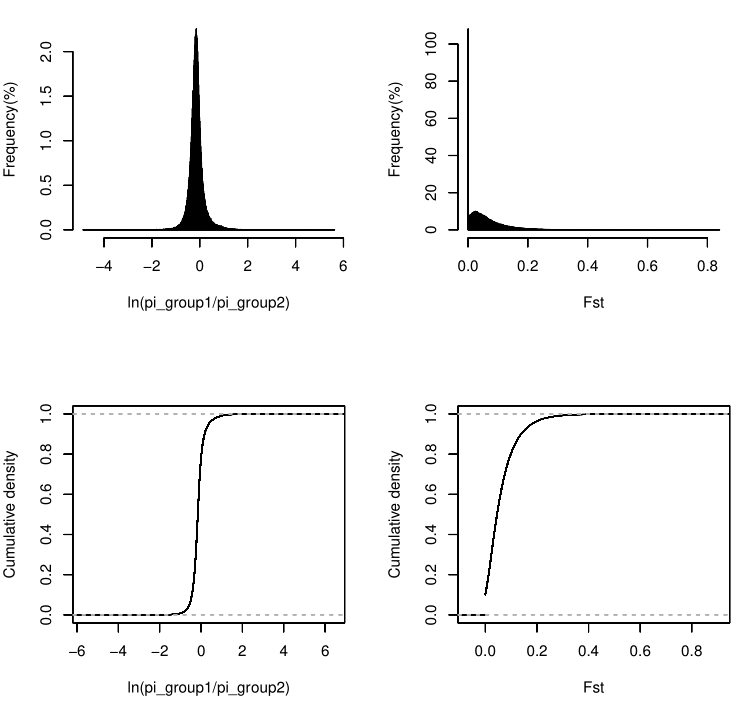

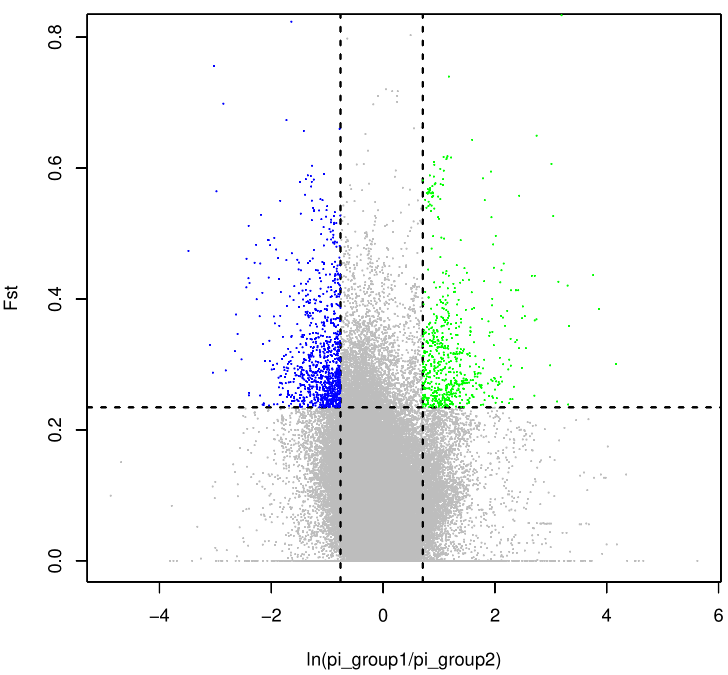


**Figure S7. The distribution of pi and FST of group 1 and wild yaks.**


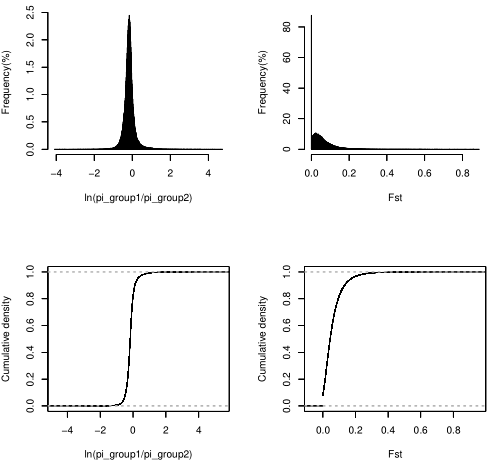

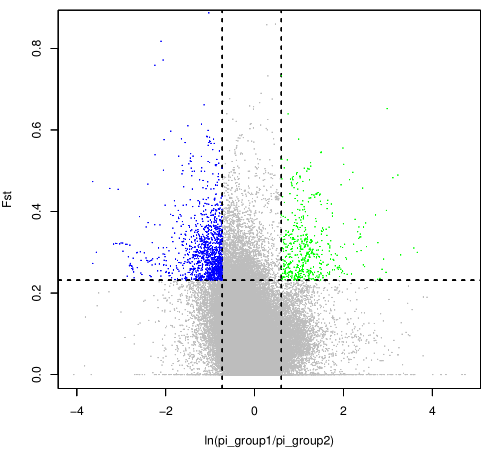


**Figure S8. The distribution of pi and FST of group 2 and wild yaks.**


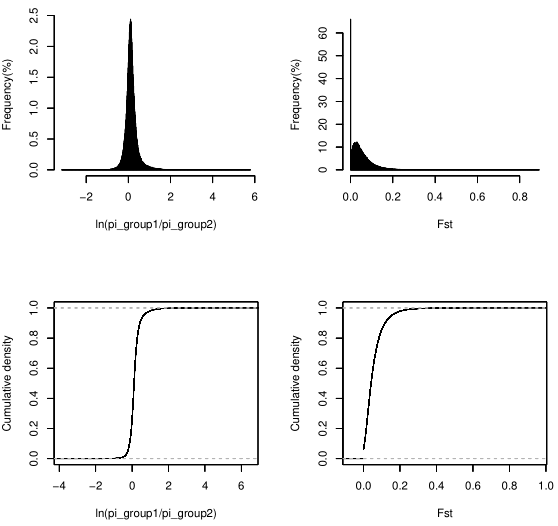

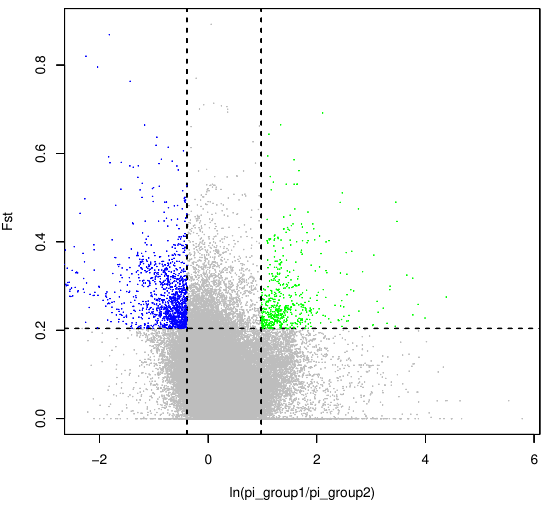


**Figure S9. The distribution of pi and FST of group 3 and wild yaks.**


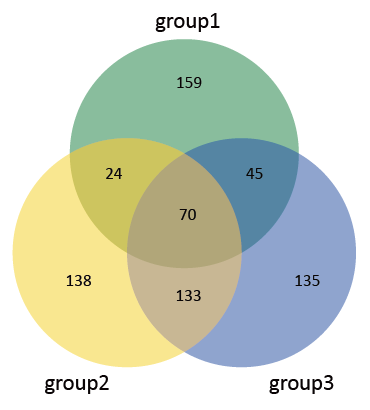


**Figure S10. The Venn diagram of selective-sweep region-related genes in the three groups.**

**Table S1. Sample information of each individual from NCBI used for resequencing.**

| Run | Sample | Base(Mbp) |
| --- | --- | --- |
| SRR2059948 | WYX01 | 14869 |
| SRR2059949 | WYX02 | 15642 |
| SRR2059950 | WYX03 | 14529 |
| SRR2059951 | WYX04 | 15995 |
| SRR2059952 | WYX05 | 21766 |
| SRR2059961 | WYX06 | 22234 |
| SRR2059962 | WYX09 | 24039 |
| SRR2059963 | WYX15 | 20831 |
| SRR2059964 | WYX16 | 18479 |
| SRR2059965 | WYX17 | 21805 |
| SRR2059966 | WYX18 | 66801 |
| SRR2059968 | WYX29 | 28269 |
| SRR2059969 | WYX31 | 71161 |
| SRR2059970 | WYX27 | 19287 |
| SRR2062306 | WYX20 | 57281 |

**Table S2. Sequence and map statistics of each individual for resequencing.**

| Sample | position | Reads number | Base | Mapped reads | Uniq map | Depth | Map ratio |
| --- | --- | --- | --- | --- | --- | --- | --- |
| BQ203 | Baqing | 90685430 | 13602814500 | 72297236 | 71350326 | 5.119614038 | 79.72% |
| BQ223 | Baqing | 92356616 | 13853492400 | 73426424 | 72406322 | 5.213960256 | 79.50% |
| BQ241 | Baqing | 91099432 | 13664914800 | 72736918 | 71765464 | 5.142986376 | 79.84% |
| CN18 | Cuona | 92643928 | 13896589200 | 71125806 | 70052250 | 5.230180354 | 76.77% |
| CN19 | Cuona | 92655500 | 13898325000 | 70114663 | 69017710 | 5.230833647 | 75.67% |
| CN4 | Cuona | 89928456 | 13489268400 | 68670131 | 67645659 | 5.076879338 | 76.36% |
| CT_43 | Changtai | 91050324 | 13657548600 | 67631269 | 66701403 | 5.140214001 | 74.28% |
| CT_59 | Changtai | 90953056 | 13642958400 | 67995836 | 67038326 | 5.13472277 | 74.76% |
| CT_92 | Changtai | 91459960 | 13718994000 | 68256404 | 67256020 | 5.163339857 | 74.63% |
| DQ251 | Dingqing | 91055388 | 13658308200 | 68037798 | 67042452 | 5.140499887 | 74.72% |
| DQ252 | Dingqing | 89559182 | 13523436482 | 68571167 | 67561801 | 5.089738985 | 76.57% |
| DQ258 | Dingqing | 89298336 | 13484048736 | 71487026 | 70503273 | 5.074914842 | 80.05% |
| GD394 | Gongbo'gyamda | 92040498 | 13898115198 | 68938293 | 67913388 | 5.230754685 | 74.90% |
| GD423 | Gongbo'gyamda | 91831652 | 13866579452 | 73856274 | 72864871 | 5.218885755 | 80.43% |
| GD424 | Gongbo'gyamda | 90874138 | 13721994838 | 72229496 | 71278630 | 5.164469265 | 79.48% |
| JC_13 | Jinchuang | 89690214 | 13543222314 | 68521953 | 67507414 | 5.097185666 | 76.40% |
| JC_15 | Jinchuang | 90144886 | 13611877786 | 68498514 | 67490953 | 5.123025136 | 75.99% |
| JC_17 | Jinchuang | 89848648 | 13567145848 | 68699341 | 67660187 | 5.10618963 | 76.46% |
| JD289 | Jiangda | 92039146 | 13897911046 | 74930163 | 73959557 | 5.230677849 | 81.41% |
| JD306 | Jiangda | 92479986 | 13964477886 | 75109273 | 74167644 | 5.255731233 | 81.22% |
| JD331 | Jiangda | 90153166 | 13613128066 | 73042621 | 72113355 | 5.123495697 | 81.02% |
| KB120 | Kangbu | 88986898 | 13437021598 | 68533341 | 67544991 | 5.057215505 | 77.02% |
| KB123 | Kangbu | 91909132 | 13878278932 | 70494620 | 69462587 | 5.223289022 | 76.70% |
| KB150 | Kangbu | 91290588 | 13784878788 | 73562130 | 72570039 | 5.18813654 | 80.58% |
| LZ29 | Longzi | 89598926 | 13529437826 | 67850340 | 66813922 | 5.091997676 | 75.73% |
| LZ30 | Longzi | 88930000 | 13428430000 | 67574465 | 66550039 | 5.053981935 | 75.99% |
| LZ49 | Longzi | 90670450 | 13691237950 | 71858815 | 70848637 | 5.15289347 | 79.25% |
| N1 | Geermu | 88893808 | 13422965008 | 67364451 | 66406012 | 5.051925107 | 75.78% |
| N2 | Geermu | 91136398 | 13670459700 | 68976731 | 67957020 | 5.145073278 | 75.69% |
| N3 | Geermu | 89880910 | 13482136500 | 67470524 | 66456964 | 5.074195145 | 75.07% |
| NR3 | Nyainrong | 91454228 | 13809588428 | 69132239 | 68102238 | 5.197436367 | 75.59% |
| NR42 | Nyainrong | 90099218 | 13604981918 | 69470428 | 68489455 | 5.120429777 | 77.10% |
| NR50 | Nyainrong | 90915442 | 13728231742 | 70751053 | 69790915 | 5.166816613 | 77.82% |
| QL1 | Qilian | 90488478 | 13663760178 | 68814051 | 67810161 | 5.142551817 | 76.05% |
| QL2 | Qilian | 89970408 | 13495561200 | 67561962 | 66552462 | 5.079247723 | 75.09% |
| QL3 | Qilian | 92875758 | 13931363700 | 70391580 | 69360963 | 5.243268235 | 75.79% |
| RD20 | Riduo | 91096030 | 13755500530 | 71121870 | 70158287 | 5.177079612 | 78.07% |
| RD25 | Riduo | 90949550 | 13733382050 | 69723741 | 68696569 | 5.168755006 | 76.66% |
| RD30 | Riduo | 92255348 | 13930557548 | 71708776 | 70670084 | 5.242964828 | 77.73% |
| SR336 | Sangri | 89719126 | 13547588026 | 72341939 | 71393257 | 5.098828764 | 80.63% |
| SR341 | Sangri | 91888580 | 13875175580 | 73792172 | 72816228 | 5.222121031 | 80.31% |
| SR343 | Sangri | 88305712 | 13334162512 | 71796633 | 70906985 | 5.018503015 | 81.30% |
| SS15 | Sangsang | 91966320 | 13886914320 | 74138044 | 73153196 | 5.226539074 | 80.61% |
| SS44 | Sangsang | 90452456 | 13658320856 | 61105922 | 60223504 | 5.14050465 | 67.56% |
| SS6 | Sangsang | 89407738 | 13500568438 | 71365013 | 70412076 | 5.081132269 | 79.82% |
| SZ13 | Shenzha | 88372892 | 13344306692 | 61055549 | 60077608 | 5.022320923 | 69.09% |
| SZ4 | Shenzha | 91714324 | 13848862924 | 73305572 | 72357070 | 5.212217886 | 79.93% |
| SZ78 | Shenzha | 91855882 | 13870238182 | 68349871 | 67294705 | 5.220262771 | 74.41% |
| ZB14 | Zhongba | 90038556 | 13595821956 | 68744006 | 67740144 | 5.116982294 | 76.35% |
| ZB27 | Zhongba | 88876762 | 13420391062 | 72739287 | 71823552 | 5.050956365 | 81.84% |
| ZB3 | Zhongba | 89053158 | 13447026858 | 62451520 | 61493854 | 5.060981128 | 70.13% |
| ZD_4 | Zhongdian | 89628400 | 13444260000 | 69483683 | 68590396 | 5.059939782 | 77.52% |
| ZD_5 | Zhongdian | 89551170 | 13432675500 | 67525477 | 66611054 | 5.055579789 | 75.40% |
| ZD_6 | Zhongdian | 91554632 | 13733194800 | 71988671 | 71087491 | 5.168684531 | 78.63% |
| BZ1 | Bazhou | 182775362 | 27599079662 | 137086451 | 135111432 | 10.38730887 | 75.00% |
| BZ2 | Bazhou | 176407204 | 26637487804 | 130746280 | 128867360 | 10.0254 | 74.12% |
| BZ3 | Bazhou | 180026730 | 27184036230 | 131944473 | 130104623 | 10.23110133 | 73.29% |
| TZ1 | Tianzu | 180105234 | 27195890334 | 121589611 | 120142778 | 10.23556279 | 67.51% |
| TZ2 | Tianzu | 177588384 | 26815845984 | 134355301 | 132422368 | 10.09252766 | 75.66% |
| TZ3 | Tianzu | 177096376 | 26741552776 | 132354542 | 130418976 | 10.06456634 | 74.74% |
| SB1269 | Sibu | 178223642 | 26911769942 | 144128573 | 142377039 | 10.12863001 | 80.87% |
| SB1289 | Sibu | 184761666 | 27899011566 | 147489668 | 145639389 | 10.50019254 | 79.83% |
| SB1294 | Sibu | 178559048 | 26962416248 | 145251429 | 143411360 | 10.14769147 | 81.35% |
| PL020 | Pali | 177823468 | 26851343668 | 140969325 | 139067795 | 10.10588772 | 79.27% |
| PL440 | Pali | 179506784 | 27105524384 | 143397054 | 141514516 | 10.20155227 | 79.88% |
| PL9 | Pali | 183941624 | 27775185224 | 137849668 | 135714637 | 10.45358872 | 74.94% |
| MW1 | Maiwa | 181721332 | 27439921132 | 130259705 | 128514499 | 10.32740728 | 71.68% |
| MW2 | Maiwa | 182948888 | 27625282088 | 132273953 | 130397142 | 10.39717053 | 72.30% |
| MW3 | Maiwa | 183263212 | 27672745012 | 136064060 | 134115381 | 10.41503388 | 74.25% |
| LWQ46 | Riwoqê | 184064908 | 27793801108 | 144243850 | 142297417 | 10.46059507 | 78.37% |
| LWQ47 | Riwoqê | 181265432 | 27371080232 | 136123612 | 134235608 | 10.30149802 | 75.10% |
| LWQ5 | Riwoqê | 184305760 | 27830169760 | 139176951 | 137192165 | 10.47428294 | 75.51% |
| JL170 | Niangya | 184299632 | 27829244432 | 140463092 | 138531037 | 10.47393468 | 76.21% |
| JL185a | Niangya | 184748960 | 27897092960 | 148379163 | 146631823 | 10.49947044 | 80.31% |
| JL185 | Niangya | 184191630 | 27812936130 | 138560286 | 136622665 | 10.46779681 | 75.23% |
| JL1 | Jiulong | 180347706 | 27232503606 | 130874718 | 129054386 | 10.24934272 | 72.57% |
| JL2 | Jiulong | 177420652 | 26790518452 | 125702709 | 123946851 | 10.08299528 | 70.85% |
| JL3 | Jiulong | 180440124 | 27246458724 | 132342435 | 130518358 | 10.25459493 | 73.34% |
| GN1 | Gannan | 177261772 | 26766527572 | 126645119 | 125006543 | 10.07396597 | 71.45% |
| GN2 | Gannan | 177424694 | 26791128794 | 130290916 | 128498987 | 10.08322499 | 73.43% |
| GN3 | Gannan | 182434882 | 27547667182 | 133889733 | 132044140 | 10.36795904 | 73.39% |
| GY31 | Gaoyuan | 179766742 | 27144778042 | 137780965 | 135841684 | 10.21632595 | 76.64% |
| GY3 | Gaoyuan | 183748462 | 27746017762 | 137823224 | 135920315 | 10.44261113 | 75.01% |
| GY9 | Gaoyuan | 176164066 | 26600773966 | 132771733 | 130902921 | 10.01158222 | 75.37% |
| HH1 | Huanghu | 183103884 | 27648686484 | 130394206 | 128721366 | 10.40597911 | 71.21% |
| HH2 | Huanghu | 183588184 | 27721815784 | 134041884 | 132257646 | 10.43350237 | 73.01% |
| HH3 | Huanghu | 181441950 | 27397734450 | 135911666 | 133947692 | 10.31152971 | 74.91% |
| DT1 | Datong | 176204314 | 26606851414 | 131862333 | 130020573 | 10.01386956 | 74.83% |
| DT2 | Datong | 184015196 | 27786294596 | 140354032 | 138273614 | 10.45776989 | 76.27% |
| DT3 | Datong | 182973130 | 27628942630 | 133498474 | 131632587 | 10.39854822 | 72.96% |
| Total |  | 11409596254 | 1,721,300,548,588 | 8663612276 | 8543437143 | 7.198179 | 75.93% |

**Table S3. nucleotide diversity of each individual.**

| Sample | Pi | Theta |
| --- | --- | --- |
| DT | 0.001640645 | 0.00165543 |
| GY | 0.001638363 | 0.001646973 |
| LWQ | 0.001638115 | 0.00165026 |
| SB | 0.001629298 | 0.00164058 |
| Jiali | 0.001626734 | 0.001627484 |
| HH | 0.001616002 | 0.001622752 |
| BZ | 0.001612189 | 0.001618267 |
| GN | 0.001609476 | 0.001612064 |
| MW | 0.001606963 | 0.00161653 |
| TZ | 0.001606938 | 0.001603487 |
| PL | 0.001574557 | 0.001588022 |
| XJ | 0.001533011 | 0.001556482 |
| Jiulong | 0.001458794 | 0.00145731 |
| BQ | 0.001207448 | 0.001196178 |
| JD | 0.001207266 | 0.001194481 |
| SR | 0.001198693 | 0.001181614 |
| Wild | 0.001187821 | 0.001134368 |
| GD | 0.001187742 | 0.001170005 |
| RD | 0.001183269 | 0.001175068 |
| NR | 0.001172228 | 0.001159718 |
| N | 0.001156587 | 0.00114205 |
| JC | 0.001156442 | 0.001142011 |
| QL | 0.001151144 | 0.001143102 |
| CT | 0.001150477 | 0.001136597 |
| SZ | 0.001149985 | 0.001117379 |
| ZD | 0.001148938 | 0.001145676 |
| SS | 0.001146243 | 0.001146508 |
| ZB | 0.001137509 | 0.001114028 |
| DQ | 0.00113033 | 0.001120002 |
| KB | 0.001125033 | 0.001111171 |
| LZ | 0.001118834 | 0.001107728 |
| CN | 0.001046995 | 0.001056127 |

**Table S4. FST statistics of each group.**

| Type | F st |
| --- | --- |
| group1-vs-group2 | 0.0275722 |
| group1-vs-group3 | 0.020518 |
| group1-vs-group_wild | 0.0684411 |
| group2-vs-group3 | 0.0189357 |
| group2-vs-group_wild | 0.0654729 |
| group3-vs-group_wild | 0.0587077 |

**Table S5. The top 0.1% FST region-related genes of groups 1–3 and the wild yak group.**

| Group | Gene | Fst | geneid | pruduct |
| --- | --- | --- | --- | --- |
| 1 | Bmu010523.1 | 0.481067 | LOC102269884 | pituitary homeobox homolog Ptx1-like |
|  | Bmu010524.1 | 0.481067 | CATSPER3 | cation channel sperm-associated protein 3 |
|  | Bmu013259.3 | 0.487694 | PRPS2 | ribose-phosphate pyrophosphokinase 2 isoform X1 |
|  | Bmu012966.1 | 0.486031 | SOX2 | transcription factor SOX-2 |
|  | Bmu017088.1 | 0.510927 | USP40 | ubiquitin carboxyl-terminal hydrolase 40 |
|  | Bmu017089.1 | 0.510927 | DGKD | LOW QUALITY PROTEIN: diacylglycerol kinase delta |
|  | Bmu017432.1 | 0.516321 | XKR8 | XK-related protein 8 |
|  | Bmu017431.1 | 0.516321 | SMPDL3B | acid sphingomyelinase-like phosphodiesterase 3b |
|  | Bmu017429.1 | 0.516321 | THEMIS2 | protein THEMIS2 |
|  | Bmu017430.1 | 0.516321 | RPA2 | replication protein A 32 kDa subunit |
|  | Bmu018017.1 | 0.486334 | EXOSC7 | exosome complex component RRP42 |
|  | Bmu018016.1 | 0.486334 | CLEC3B | tetranectin |
|  | Bmu018018.1 | 0.486334 | ZDHHC3 | palmitoyltransferase ZDHHC3 isoform X2 |
|  | Bmu018278.1 | 0.483286 | ZNF259 | zinc finger protein ZPR1 |
|  | Bmu018279.1 | 0.483286 | APOA5 | apolipoprotein A-V |
|  | Bmu018277.1 | 0.483286 | BUD13 | BUD13 homolog isoform X1 |
|  | Bmu018586.1 | 0.491628 | NAT8L | N-acetylaspartate synthetase |
|  | Bmu018585.1 | 0.491628 | LOC102287324 | basic proline-rich protein-like |
|  | Bmu018587.1 | 0.491628 | NELFA | negative elongation factor A |
|  | Bmu001516.1 | 0.530927 | PAIP2 | polyadenylate-binding protein-interacting protein 2 |
|  | Bmu001515.1 | 0.530927 | MATR3 | matrin-3-like isoform X4 |
|  | Bmu001517.1 | 0.530927 | SLC23A1 | solute carrier family 23 member 1 isoform X3 |
|  | Bmu002268.1 | 0.506949 | TNFSF15 | tumor necrosis factor ligand superfamily member 15 |
|  | Bmu004053.1 | 0.560499 | LOC102279949 | histone deacetylase complex subunit SAP18-like |
|  | Bmu004054.1 | 0.560499 | PACSIN2 | protein kinase C and casein kinase substrate in neurons protein 2 |
|  | Bmu005869.1 | 0.698374 | LOC102277803 | boLa class II histocompatibility antigen%2C DQB%2A0101 beta chain-like |
|  | Bmu005750.1 | 0.510528 | PLA2G2E | group IIE secretory phospholipase A2 |
|  | Bmu005751.1 | 0.510528 | OTUD3 | OTU domain-containing protein 3 |
|  | Bmu005749.1 | 0.510528 | LOC102276213 | phospholipase A2%2C membrane associated-like |
|  | Bmu009745.3 | 0.483908 | AIFM1 | apoptosis-inducing factor 1%2C mitochondrial isoform X2 |
|  | Bmu009744.1 | 0.483908 | ELF4 | ETS-related transcription factor Elf-4 |
|  | Bmu010327.1 | 0.67243 | TCIRG1 | V-type proton ATPase 116 kDa subunit a isoform 3 |
|  | Bmu010328.1 | 0.67243 | CHKA | choline kinase alpha |
|  | Bmu010329.1 | 0.67243 | SUV420H1 | histone-lysine N-methyltransferase SUV420H1 |
|  | Bmu010326.1 | 0.67243 | NDUFS8 | NADH dehydrogenase %5Bubiquinone%5D iron-sulfur protein 8%2C mitochondrial isoform X2 |
|  | Bmu010325.1 | 0.67243 | ALDH3B1 | aldehyde dehydrogenase family 3 member B1 |
|  | Bmu000015.1 | 0.649544 | LOC102284589 | hemoglobin subunit zeta-like |
| 2 | Bmu018722.1 | 0.516935 | PLAGL2 | zinc finger protein PLAGL2 |
|  | Bmu018724.1 | 0.516935 | HCK | tyrosine-protein kinase HCK isoform X1 |
|  | Bmu018723.1 | 0.516935 | TM9SF4 | transmembrane 9 superfamily member 4 isoform X1 |
|  | Bmu010518.1 | 0.494399 | SLC25A48 | solute carrier family 25 member 48 |
|  | Bmu010516.1 | 0.494399 | FBXL21 | F-box%2FLRR-repeat protein 21 |
|  | Bmu010517.1 | 0.494399 | IL9 | interleukin-9 |
|  | Bmu010515.1 | 0.494399 | LECT2 | leukocyte cell-derived chemotaxin-2 |
|  | Bmu010523.1 | 0.491573 | LOC102269884 | pituitary homeobox homolog Ptx1-like |
|  | Bmu010524.1 | 0.491573 | CATSPER3 | cation channel sperm-associated protein 3 |
|  | Bmu017432.1 | 0.552944 | XKR8 | XK-related protein 8 |
|  | Bmu017431.1 | 0.552944 | SMPDL3B | acid sphingomyelinase-like phosphodiesterase 3b |
|  | Bmu017429.1 | 0.552944 | THEMIS2 | protein THEMIS2 |
|  | Bmu017430.1 | 0.552944 | RPA2 | replication protein A 32 kDa subunit |
|  | Bmu018586.1 | 0.506046 | NAT8L | N-acetylaspartate synthetase |
|  | Bmu018585.1 | 0.506046 | LOC102287324 | basic proline-rich protein-like |
|  | Bmu018587.1 | 0.506046 | NELFA | negative elongation factor A |
|  | Bmu000315.1 | 0.54484 | LOC102266460 | 60S ribosomal protein L23a-like |
|  | Bmu001284.1 | 0.501787 | LOC102276483 | acid-sensing ion channel 2-like |
|  | Bmu002267.1 | 0.537921 | TNFSF8 | tumor necrosis factor ligand superfamily member 8 |
|  | Bmu002268.1 | 0.537921 | TNFSF15 | tumor necrosis factor ligand superfamily member 15 |
|  | Bmu004053.1 | 0.507557 | LOC102279949 | histone deacetylase complex subunit SAP18-like |
|  | Bmu004054.1 | 0.507557 | PACSIN2 | protein kinase C and casein kinase substrate in neurons protein 2 |
|  | Bmu007246.1 | 0.479303 | RAC3 | ras-related C3 botulinum toxin substrate 3 |
|  | Bmu007250.1 | 0.479303 | NOTUM | protein notum homolog |
|  | Bmu007247.1 | 0.479303 | LRRC45 | leucine-rich repeat-containing protein 45 |
|  | Bmu007244.1 | 0.479303 | LOC102279110 | carbonyl reductase %5BNADPH%5D 2-like |
|  | Bmu007249.1 | 0.479303 | ASPSCR1 | tether containing UBX domain for GLUT4 |
|  | Bmu007251.1 | 0.479303 | MYADML2 | myeloid-associated differentiation marker-like protein 2 |
|  | Bmu007245.1 | 0.479303 | DCXR | L-xylulose reductase |
|  | Bmu007252.2 | 0.479303 | PYCR1 | pyrroline-5-carboxylate reductase 1%2C mitochondrial isoform X1 |
|  | Bmu007248.1 | 0.479303 | STRA13 | centromere protein X |
|  | Bmu007201.1 | 0.501712 | LOC102276782 | histone H2B type F-M-like |
|  | Bmu007200.1 | 0.501712 | RGAG1 | retrotransposon gag domain-containing protein 1 |
|  | Bmu008776.2 | 0.490273 | NRM | nurim isoform X1 |
|  | Bmu008767.1 | 0.490273 | GNL1 | LOW QUALITY PROTEIN: guanine nucleotide-binding protein-like 1 |
|  | Bmu008771.1 | 0.490273 | MRPS18B | 28S ribosomal protein S18b%2C mitochondrial |
|  | Bmu008766.1 | 0.490273 | RPP21 | ribonuclease P protein subunit p21 |
|  | Bmu008772.1 | 0.490273 | ATAT1 | alpha-tubulin N-acetyltransferase isoform X1 |
|  | Bmu008774.1 | 0.490273 | DHX16 | LOW QUALITY PROTEIN: putative pre-mRNA-splicing factor ATP-dependent RNA helicase DHX16 |
|  | Bmu008765.1 | 0.490273 | TRIM39 | E3 ubiquitin-protein ligase TRIM39 |
|  | Bmu008775.1 | 0.490273 | PPP1R18 | phostensin |
|  | Bmu008769.1 | 0.490273 | ABCF1 | ATP-binding cassette sub-family F member 1 isoform X1 |
|  | Bmu008768.1 | 0.490273 | PRR3 | proline-rich protein 3 isoform X1 |
|  | Bmu008773.1 | 0.490273 | LOC102276225 | uncharacterized protein C6orf136 homolog |
|  | Bmu008770.1 | 0.490273 | PPP1R10 | LOW QUALITY PROTEIN: protein phosphatase 1%2C regulatory subunit 10 |
|  | Bmu008777.1 | 0.490273 | MDC1 | LOW QUALITY PROTEIN: mediator of DNA damage checkpoint protein 1 |
|  | Bmu009987.1 | 0.614491 | PROSER1 | proline and serine-rich protein 1 |
|  | Bmu009986.1 | 0.614491 | NHLRC3 | NHL repeat-containing protein 3 |
|  | Bmu010327.1 | 0.57651 | TCIRG1 | V-type proton ATPase 116 kDa subunit a isoform 3 |
|  | Bmu010328.1 | 0.57651 | CHKA | choline kinase alpha |
|  | Bmu010329.1 | 0.57651 | SUV420H1 | histone-lysine N-methyltransferase SUV420H1 |
|  | Bmu010326.1 | 0.57651 | NDUFS8 | NADH dehydrogenase %5Bubiquinone%5D iron-sulfur protein 8%2C mitochondrial isoform X2 |
|  | Bmu010325.1 | 0.57651 | ALDH3B1 | aldehyde dehydrogenase family 3 member B1 |
|  | Bmu011976.1 | 0.523939 | LOC102274355 | olfactory receptor 4C16-like |
|  | Bmu014739.1 | 0.492367 | LOC102274457 | secretoglobin family 2B member 2-like |
|  | Bmu000015.1 | 0.515578 | LOC102284589 | hemoglobin subunit zeta-like |
| 3 | Bmu018725.1 | 0.541252 | CCM2L | LOW QUALITY PROTEIN: cerebral cavernous malformation 2-like |
|  | Bmu018722.1 | 0.541252 | PLAGL2 | zinc finger protein PLAGL2 |
|  | Bmu018724.1 | 0.541252 | HCK | tyrosine-protein kinase HCK isoform X1 |
|  | Bmu018723.1 | 0.541252 | TM9SF4 | transmembrane 9 superfamily member 4 isoform X1 |
|  | Bmu010518.1 | 0.448851 | SLC25A48 | solute carrier family 25 member 48 |
|  | Bmu010516.1 | 0.448851 | FBXL21 | F-box%2FLRR-repeat protein 21 |
|  | Bmu010517.1 | 0.448851 | IL9 | interleukin-9 |
|  | Bmu010515.1 | 0.448851 | LECT2 | leukocyte cell-derived chemotaxin-2 |
|  | Bmu017432.1 | 0.477414 | XKR8 | XK-related protein 8 |
|  | Bmu017431.1 | 0.477414 | SMPDL3B | acid sphingomyelinase-like phosphodiesterase 3b |
|  | Bmu017429.1 | 0.477414 | THEMIS2 | protein THEMIS2 |
|  | Bmu017430.1 | 0.477414 | RPA2 | replication protein A 32 kDa subunit |
|  | Bmu000315.1 | 0.454243 | LOC102266460 | 60S ribosomal protein L23a-like |
|  | Bmu001284.1 | 0.475557 | LOC102276483 | acid-sensing ion channel 2-like |
|  | Bmu002267.1 | 0.490353 | TNFSF8 | tumor necrosis factor ligand superfamily member 8 |
|  | Bmu002268.1 | 0.490353 | TNFSF15 | tumor necrosis factor ligand superfamily member 15 |
|  | Bmu004054.1 | 0.41859 | PACSIN2 | protein kinase C and casein kinase substrate in neurons protein 2 |
|  | Bmu005750.1 | 0.497178 | PLA2G2E | group IIE secretory phospholipase A2 |
|  | Bmu005751.1 | 0.497178 | OTUD3 | OTU domain-containing protein 3 |
|  | Bmu005749.1 | 0.497178 | LOC102276213 | phospholipase A2%2C membrane associated-like |
|  | Bmu007253.1 | 0.439322 | MAFG | transcription factor MafG |
|  | Bmu007246.1 | 0.439322 | RAC3 | ras-related C3 botulinum toxin substrate 3 |
|  | Bmu007250.1 | 0.439322 | NOTUM | protein notum homolog |
|  | Bmu007247.1 | 0.439322 | LRRC45 | leucine-rich repeat-containing protein 45 |
|  | Bmu007244.1 | 0.439322 | LOC102279110 | carbonyl reductase %5BNADPH%5D 2-like |
|  | Bmu007249.1 | 0.439322 | ASPSCR1 | tether containing UBX domain for GLUT4 |
|  | Bmu007251.1 | 0.439322 | MYADML2 | myeloid-associated differentiation marker-like protein 2 |
|  | Bmu007254.1 | 0.439322 | SIRT7 | NAD-dependent protein deacetylase sirtuin-7 |
|  | Bmu007245.1 | 0.439322 | DCXR | L-xylulose reductase |
|  | Bmu007252.2 | 0.439322 | PYCR1 | pyrroline-5-carboxylate reductase 1%2C mitochondrial isoform X1 |
|  | Bmu007248.1 | 0.439322 | STRA13 | centromere protein X |
|  | Bmu009987.1 | 0.531987 | PROSER1 | proline and serine-rich protein 1 |
|  | Bmu010327.1 | 0.670058 | TCIRG1 | V-type proton ATPase 116 kDa subunit a isoform 3 |
|  | Bmu010328.1 | 0.670058 | CHKA | choline kinase alpha |
|  | Bmu010329.1 | 0.670058 | SUV420H1 | histone-lysine N-methyltransferase SUV420H1 |
|  | Bmu010326.1 | 0.670058 | NDUFS8 | NADH dehydrogenase %5Bubiquinone%5D iron-sulfur protein 8%2C mitochondrial isoform X2 |
|  | Bmu010325.1 | 0.670058 | ALDH3B1 | aldehyde dehydrogenase family 3 member B1 |
|  | Bmu011976.1 | 0.418219 | LOC102274355 | olfactory receptor 4C16-like |
|  | Bmu018941.1 | 0.532173 | LOC102285613 | zinc finger protein 160-like |

**Table S6. The enrichment of top 1% FST region-related genes between two of the three groups.**

|  | ID | Term | Class | Pvalue |
| --- | --- | --- | --- | --- |
| 1 vs 2 | GO:0002504 | antigen processing and presentation of peptide or polysaccharide antigen via MHC class II | BP | 6.30E-09 |
|  | GO:0042613 | MHC class II protein complex | CC | 1.72E-08 |
|  | GO:0030151 | molybdenum ion binding | MF | 3.11E-06 |
|  | map04940 | Type I diabetes mellitus | KEGG | 4.56E-06 |
|  | map05332 | Graft-versus-host disease | KEGG | 5.00E-06 |
|  | map05323 | Rheumatoid arthritis | KEGG | 1.70E-05 |
|  | map05310 | Asthma | KEGG | 4.04E-05 |
|  | map05330 | Allograft rejection | KEGG | 4.79E-05 |
|  | map04672 | Intestinal immune network for IgA production | KEGG | 7.72E-05 |
|  | map04612 | Antigen processing and presentation | KEGG | 8.09E-05 |
|  | map05140 | Leishmaniasis | KEGG | 9.98E-05 |
|  | map05320 | Autoimmune thyroid disease | KEGG | 0.000236308 |
|  | map04640 | Hematopoietic cell lineage | KEGG | 0.000275022 |
|  | map05416 | Viral myocarditis | KEGG | 0.000425049 |
|  | map04940 | Type I diabetes mellitus | KEGG | 4.56E-06 |
| 1 vs 3 | GO:0042613 | MHC class II protein complex | CC | 1.38E-07 |
|  | GO:0002504 | antigen processing and presentation of peptide or polysaccharide antigen via MHC class II | BP | 2.61E-06 |
|  | map04672 | Intestinal immune network for IgA production | KEGG | 4.40E-06 |
|  | map05322 | Systemic lupus erythematosus | KEGG | 2.14E-05 |
|  | map05330 | Allograft rejection | KEGG | 2.99E-05 |
|  | map04940 | Type I diabetes mellitus | KEGG | 4.81E-05 |
|  | map05332 | Graft-versus-host disease | KEGG | 5.27E-05 |
|  | map05320 | Autoimmune thyroid disease | KEGG | 0.000197 |
|  | map05310 | Asthma | KEGG | 0.000285 |
|  | map05150 | Staphylococcus aureus infection | KEGG | 0.000286 |

**Table S7. The 70 shared selective-sweep genes of the three groups.**

| Functional classification | Gene | gene id | product |
| --- | --- | --- | --- |
| \| metabolic \| \| --- \| \|  \| \|  \| \|  \| \|  \| \|  \| \|  \| \|  \| \|  \| \|  \| \|  \| \|  \| \|  \| \|  \| \|  \| \|  \| \|  \| \|  \| | Bmu001226.1 | LOC102266101 | 40S ribosomal protein S15a-like |
|  | Bmu004053.1 | LOC102279949 | histone deacetylase complex subunit SAP18-like |
|  | Bmu004076.1 | LOC102276035 | zinc finger protein 316-like |
|  | Bmu004077.1 | ZNF674 | zinc finger protein 674-like |
|  | Bmu005352.1 | KDM2A | lysine-specific demethylase 2A |
|  | Bmu006185.1 | LOC102284536 | 6-phosphofructo-2-kinase%2Ffructose-2%2C6-bisphosphatase 1-like |
|  | Bmu007265.1 | MRPL12 | 39S ribosomal protein L12%2C mitochondrial |
|  | Bmu007275.1 | ACTG1 | actin%2C cytoplasmic 2 |
|  | Bmu008055.1 | MAFA | LOW QUALITY PROTEIN: transcription factor MafA |
|  | Bmu008057.1 | TOP1MT | LOW QUALITY PROTEIN: DNA topoisomerase I%2C mitochondrial |
|  | Bmu017258.1 | APEX2 | DNA-%28apurinic or apyrimidinic site%29 lyase 2 isoform X1 |
|  | Bmu017259.1 | PFKFB1 | 6-phosphofructo-2-kinase%2Ffructose-2%2C6-bisphosphatase 1 |
|  | Bmu017431.1 | SMPDL3B | acid sphingomyelinase-like phosphodiesterase 3b |
|  | Bmu017518.1 | LOC102284418 | asparagine--tRNA ligase%2C cytoplasmic-like |
|  | Bmu018722.1 | PLAGL2 | zinc finger protein PLAGL2 |
|  | Bmu018724.1 | HCK | tyrosine-protein kinase HCK isoform X1 |
|  | Bmu018737.1 | HM13 | minor histocompatibility antigen H13 isoform X1 |
|  | Bmu018738.1 | REM1 | GTP-binding protein REM 1 |
| \| nerve/organ development \| \| --- \| \|  \| \|  \| \|  \| \|  \| \|  \| \|  \| \|  \| \|  \| | Bmu018022.1 | ATP2B2 | plasma membrane calcium-transporting ATPase 2 |
|  | Bmu015807.1 | CACNA1B | voltage-dependent N-type calcium channel subunit alpha-1B-like |
|  | Bmu001284.1 | LOC102276483 | acid-sensing ion channel 2-like |
|  | Bmu018020.1 | GHRL | appetite-regulating hormone isoform X1 |
|  | Bmu007274.1 | FSCN2 | fascin-2 |
|  | Bmu004470.1 | BCOR | LOW QUALITY PROTEIN: BCL-6 corepressor |
|  | Bmu012019.1 | LOC102271681 | Y-box-binding protein 3-like |
|  | Bmu018721.1 | POFUT1 | GDP-fucose protein O-fucosyltransferase 1 isoform X2 |
|  | Bmu017433.2 | EYA3 | eyes absent homolog 3 isoform X3 |
| phagocytes and response to stimulus | Bmu004052.1 | ARFGAP3 | ADP-ribosylation factor GTPase-activating protein 3 isoform X1 |
|  | Bmu007266.1 | HGS | hepatocyte growth factor-regulated tyrosine kinase substrate |
|  | Bmu007268.1 | CCDC137 | coiled-coil domain-containing protein 137 |
|  | Bmu008054.1 | ZC3H3 | zinc finger CCCH domain-containing protein 3 |
|  | Bmu007270.1 | PDE6G | retinal rod rhodopsin-sensitive cGMP 3%27%2C5%27-cyclic phosphodiesterase subunit gamma |
|  | Bmu007149.1 | GPR158 | probable G-protein coupled receptor 158 |
|  | Bmu017430.1 | RPA2 | replication protein A 32 kDa subunit |
|  | Bmu017257.2 | ALAS2 | 5-aminolevulinate synthase%2C erythroid-specific%2C mitochondrial isoform X1 |
| Other | Bmu000129.1 | LOC102269272 | olfactory receptor 11L1-like |
|  | Bmu001227.1 | AP1G1 | AP-1 complex subunit gamma-1 isoform X2 |
|  | Bmu001228.1 | PHLPP2 | PH domain leucine-rich repeat-containing protein phosphatase 2 |
|  | Bmu004054.1 | PACSIN2 | protein kinase C and casein kinase substrate in neurons protein 2 |
|  | Bmu004570.1 | FAM53B | protein FAM53B |
|  | Bmu004816.1 | CAND2 | cullin-associated NEDD8-dissociated protein 2 |
|  | Bmu004817.1 | TMEM40 | transmembrane protein 40 |
|  | Bmu005351.1 | RHOD | rho-related GTP-binding protein RhoD |
|  | Bmu005868.1 | DENND6B | protein DENND6B |
|  | Bmu006184.1 | TRO | LOW QUALITY PROTEIN: trophinin |
|  | Bmu007264.1 | SLC25A10 | mitochondrial dicarboxylate carrier |
|  | Bmu007267.1 | ARL16 | ADP-ribosylation factor-like protein 16 |
|  | Bmu007269.1 | OXLD1 | oxidoreductase-like domain-containing protein 1 |
|  | Bmu007271.1 | TSPAN10 | tetraspanin-10 |
|  | Bmu007272.1 | NPLOC4 | nuclear protein localization protein 4 homolog |
|  | Bmu007273.1 | LOC102265744 | Fanconi anemia-associated protein of 100 kDa |
|  | Bmu008056.1 | RHPN1 | rhophilin-1 |
|  | Bmu010347.1 | ANO1 | anoctamin-1 |
|  | Bmu010518.1 | SLC25A48 | solute carrier family 25 member 48 |
|  | Bmu012017.1 | ZXDB | zinc finger X-linked protein ZXDB |
|  | Bmu012018.1 | LOC102271404 | zinc finger X-linked protein ZXDB-like |
|  | Bmu012020.1 | SPIN4 | spindlin-4 |
|  | Bmu012709.1 | LOC102278975 | olfactory receptor 6M1-like |
|  | Bmu014739.1 | LOC102274457 | secretoglobin family 2B member 2-like |
|  | Bmu017432.1 | XKR8 | XK-related protein 8 |
|  | Bmu017517.1 | TANGO6 | transport and Golgi organization protein 6 homolog |
|  | Bmu018019.1 | TMEM42 | transmembrane protein 42 |
|  | Bmu018021.1 | SEC13 | protein SEC13 homolog |
|  | Bmu018723.1 | TM9SF4 | transmembrane 9 superfamily member 4 isoform X1 |
|  | Bmu018725.1 | CCM2L | LOW QUALITY PROTEIN: cerebral cavernous malformation 2-like |
|  | Bmu018726.1 | XKR7 | XK-related protein 7 |
|  | Bmu018739.1 | DEFB121 | beta-defensin 121 |
|  | Bmu018941.1 | LOC102285613 | zinc finger protein 160-like |
|  | Bmu019560.1 | ICA1 | islet cell autoantigen 1 isoform X1 |
|  | Bmu001226.1 | LOC102266101 | 40S ribosomal protein S15a-like |

**Table S8. The 24 shared selective-sweep genes in wild yaks.**

| Functional classification | Gene | gene id | product |
| --- | --- | --- | --- |
| Disease, immunity | Bmu011831.1 | LOC102277558 | claudin-10-like |
|  | Bmu014503.1 | LOC102276165 | 5-hydroxyisourate hydrolase-like |
|  | Bmu014506.1 | LOC102276729 | tigger transposable element-derived protein 1-like |
|  | Bmu014508.1 | TUBB3 | tubulin beta-3 chain |
|  | Bmu014509.1 | LOC102277577 | uncharacterized protein LOC102277577 |
|  | Bmu013258.1 | FRMPD4 | FERM and PDZ domain-containing protein 4 |
|  | Bmu004717.1 | LOC102268170 | BOLA class I histocompatibility antigen%2C alpha chain BL3-7-like |
|  | Bmu007956.1 | LOC102272986 | mast%2Fstem cell growth factor receptor Kit-like isoform X2 |
|  | Bmu014512.1 | FANCA | Fanconi anemia group A protein |
|  | Bmu000015.1 | LOC102284589 | hemoglobin subunit zeta-like |
|  | Bmu014507.1 | DEF8 | differentially expressed in FDCP 8 homolog |
|  | Bmu004794.1 | LPIN1 | phosphatidate phosphatase LPIN1 isoform X2 |
| Other | Bmu001514.1 | SIL1 | nucleotide exchange factor SIL1 |
|  | Bmu003911.1 | LOC102280321 | multidrug resistance-associated protein 4-like |
|  | Bmu008216.1 | LOC102273755 | uncharacterized protein C7orf62-like |
|  | Bmu014504.1 | GAS8 | growth arrest-specific protein 8 |
|  | Bmu014505.1 | DBNDD1 | dysbindin domain-containing protein 1 |
|  | Bmu014510.1 | TCF25 | transcription factor 25 |
|  | Bmu014511.1 | SPIRE2 | protein spire homolog 2 |
|  | Bmu014513.1 | ZNF276 | zinc finger protein 276 |
|  | Bmu014514.1 | VPS9D1 | VPS9 domain-containing protein 1 |
|  | Bmu014515.1 | SPATA2L | spermatogenesis-associated protein 2-like protein |
|  | Bmu014516.1 | CDK10 | cyclin-dependent kinase 10 isoform X1 |
|  | Bmu014517.1 | SPATA33 | spermatogenesis-associated protein 33 |

**Table S9.** **The introgression statistics of each individual.**

**Table S10. The top 20 enrichment of 521 genes introgressed from yaks to Tibetan cattle.**

| ID | Term | Class | Pvalue |
| --- | --- | --- | --- |
| GO:0007155 | cell adhesion | BP | 6.34E-06 |
| GO:0022610 | biological adhesion | BP | 7.01E-06 |
| GO:0042278 | purine nucleoside metabolic process | BP | 1.89E-05 |
| GO:1901135 | carbohydrate derivative metabolic process | BP | 1.98E-05 |
| GO:0009144 | purine nucleoside triphosphate metabolic process | BP | 2.16E-05 |
| GO:0009119 | ribonucleoside metabolic process | BP | 2.68E-05 |
| GO:0009116 | nucleoside metabolic process | BP | 2.98E-05 |
| GO:0009141 | nucleoside triphosphate metabolic process | BP | 3.31E-05 |
| GO:1901657 | glycosyl compound metabolic process | BP | 3.79E-05 |
| GO:0046128 | purine ribonucleoside metabolic process | BP | 3.83E-05 |
| GO:0009164 | nucleoside catabolic process | BP | 7.79E-05 |
| GO:0072523 | purine-containing compound catabolic process | BP | 7.94E-05 |
| GO:1901658 | glycosyl compound catabolic process | BP | 8.30E-05 |
| GO:0031088 | platelet dense granule membrane | CC | 8.68E-05 |
| GO:0048639 | positive regulation of developmental growth | BP | 9.01E-05 |
| GO:0009205 | purine ribonucleoside triphosphate metabolic process | BP | 9.56E-05 |
| GO:1901136 | carbohydrate derivative catabolic process | BP | 0.000102 |
| GO:0009199 | ribonucleoside triphosphate metabolic process | BP | 0.000111 |
| GO:0042827 | platelet dense granule | CC | 0.000134 |
| GO:0051179 | localization | BP | 0.000138 |
| map02010 | ABC transporters | KEGG | 0.000182 |
| map04514 | Cell adhesion molecules (CAMs) | KEGG | 0.000317 |
| map04976 | Bile secretion | KEGG | 0.000726 |
| map04961 | Endocrine and other factor-regulated calcium reabsorption | KEGG | 0.001836 |
| map05144 | Malaria | KEGG | 0.008391 |
| map04070 | Phosphatidylinositol signaling system | KEGG | 0.00975 |
| map04971 | Gastric acid secretion | KEGG | 0.01832 |
| map00561 | Glycerolipid metabolism | KEGG | 0.024292 |
| map00523 | Polyketide sugar unit biosynthesis | KEGG | 0.026238 |
| map05143 | African trypanosomiasis | KEGG | 0.027789 |
| map05020 | Prion diseases | KEGG | 0.029612 |
| map04270 | Vascular smooth muscle contraction | KEGG | 0.031157 |
| map00563 | Glycosylphosphatidylinositol (GPI)-anchor biosynthesis | KEGG | 0.032936 |
| map04721 | Synaptic vesicle cycle | KEGG | 0.033259 |
| map00562 | Inositol phosphate metabolism | KEGG | 0.034069 |
| map04916 | Melanogenesis | KEGG | 0.035075 |
| map05218 | Melanoma | KEGG | 0.035646 |
| map03060 | Protein export | KEGG | 0.03619 |
| map04510 | Focal adhesion | KEGG | 0.043813 |
| map04540 | Gap junction | KEGG | 0.045095 |

**Table S11. The top 20 enrichment of 129 genes introgressed from Tibetan cattle to yaks.**

| ID | Term | Class | Pvalue |
| --- | --- | --- | --- |
| GO:0003980 | UDP-glucose:glycoprotein glucosyltransferase activity | MF | 4.19E-05 |
| GO:0097359 | UDP-glucosylation | BP | 4.19E-05 |
| GO:0006011 | UDP-glucose metabolic process | BP | 4.19E-05 |
| GO:0032851 | positive regulation of Rab GTPase activity | BP | 0.000244 |
| GO:0061045 | negative regulation of wound healing | BP | 0.000249 |
| GO:0006796 | phosphate-containing compound metabolic process | BP | 0.000254 |
| GO:0032313 | regulation of Rab GTPase activity | BP | 0.000265 |
| GO:0032483 | regulation of Rab protein signal transduction | BP | 0.000265 |
| GO:0005099 | Ras GTPase activator activity | MF | 0.000272 |
| GO:0005097 | Rab GTPase activator activity | MF | 0.000287 |
| GO:0006793 | phosphorus metabolic process | BP | 0.000306 |
| GO:0042043 | neurexin family protein binding | MF | 0.000618 |
| GO:0035251 | UDP-glucosyltransferase activity | MF | 0.000618 |
| GO:0006464 | cellular protein modification process | BP | 0.000931 |
| GO:0036211 | protein modification process | BP | 0.000931 |
| GO:0046527 | glucosyltransferase activity | MF | 0.001143 |
| GO:0032320 | positive regulation of Ras GTPase activity | BP | 0.001658 |
| GO:0043412 | macromolecule modification | BP | 0.001731 |
| GO:0008081 | phosphoric diester hydrolase activity | MF | 0.002196 |
| GO:0005083 | small GTPase regulator activity | MF | 0.002359 |
| map00562 | Inositol phosphate metabolism | KEGG | 0.003056 |
| map04710 | Circadian rhythm | KEGG | 0.015161 |
| map04070 | Phosphatidylinositol signaling system | KEGG | 0.020562 |
| map04060 | Cytokine-cytokine receptor interaction | KEGG | 0.044645 |
| map04630 | Jak-STAT signaling pathway | KEGG | 0.04487 |
| map04350 | TGF-beta signaling pathway | KEGG | 0.058962 |
| map03320 | PPAR signaling pathway | KEGG | 0.06536 |
| map00514 | Other types of O-glycan biosynthesis | KEGG | 0.07601 |
| map04330 | Notch signaling pathway | KEGG | 0.08556 |
| map04974 | Protein digestion and absorption | KEGG | 0.090094 |
| map04810 | Regulation of actin cytoskeleton | KEGG | 0.103766 |
| map04530 | Tight junction | KEGG | 0.107678 |
| map04114 | Oocyte meiosis | KEGG | 0.11532 |
| map05223 | Non-small cell lung cancer | KEGG | 0.120389 |
| map05218 | Melanoma | KEGG | 0.122535 |
| map05200 | Pathways in cancer | KEGG | 0.135775 |
| map00563 | Glycosylphosphatidylinositol (GPI)-anchor biosynthesis | KEGG | 0.161459 |
| map04010 | MAPK signaling pathway | KEGG | 0.166219 |
| map03060 | Protein export | KEGG | 0.166914 |
| map03430 | Mismatch repair | KEGG | 0.166914 |

**Table S12. The top 20 enrichment of 2608 genes introgressed from domestic yak to wild yak.**

| ID | Term | Class | Pvalue |
| --- | --- | --- | --- |
| GO:0043167 | ion binding | MF | 8.58E-15 |
| GO:0051179 | localization | BP | 3.66E-14 |
| GO:0043168 | anion binding | MF | 2.06E-13 |
| GO:0010646 | regulation of cell communication | BP | 1.25E-11 |
| GO:0023051 | regulation of signaling | BP | 1.30E-11 |
| GO:0007155 | cell adhesion | BP | 2.10E-11 |
| GO:0022610 | biological adhesion | BP | 2.97E-11 |
| GO:0045202 | synapse | CC | 2.54E-10 |
| GO:0051234 | establishment of localization | BP | 2.83E-10 |
| GO:0033124 | regulation of GTP catabolic process | BP | 3.73E-10 |
| GO:0048583 | regulation of response to stimulus | BP | 5.46E-10 |
| GO:0006810 | transport | BP | 8.07E-10 |
| GO:0030030 | cell projection organization | BP | 1.01E-09 |
| GO:0044765 | single-organism transport | BP | 1.10E-09 |
| GO:0009966 | regulation of signal transduction | BP | 1.12E-09 |
| GO:0005543 | phospholipid binding | MF | 1.12E-09 |
| GO:0043087 | regulation of GTPase activity | BP | 1.46E-09 |
| GO:0044699 | single-organism process | BP | 1.50E-09 |
| GO:0009118 | regulation of nucleoside metabolic process | BP | 1.79E-09 |
| GO:0032559 | adenyl ribonucleotide binding | MF | 1.98E-09 |
| map04510 | Focal adhesion | KEGG | 9.26E-15 |
| map04512 | ECM-receptor interaction | KEGG | 1.81E-12 |
| map04723 | Retrograde endocannabinoid signaling | KEGG | 1.83E-07 |
| map05033 | Nicotine addiction | KEGG | 2.55E-07 |
| map05412 | Arrhythmogenic right ventricular cardiomyopathy (ARVC) | KEGG | 8.50E-07 |
| map04520 | Adherens junction | KEGG | 3.12E-06 |
| map05032 | Morphine addiction | KEGG | 6.54E-06 |
| map04720 | Long-term potentiation | KEGG | 7.74E-06 |
| map04728 | Dopaminergic synapse | KEGG | 1.04E-05 |
| map04360 | Axon guidance | KEGG | 1.31E-05 |
| map05031 | Amphetamine addiction | KEGG | 4.00E-05 |
| map04724 | Glutamatergic synapse | KEGG | 7.86E-05 |
| map04974 | Protein digestion and absorption | KEGG | 0.000122 |
| map04070 | Phosphatidylinositol signaling system | KEGG | 0.000174 |
| map04810 | Regulation of actin cytoskeleton | KEGG | 0.000208 |
| map05146 | Amoebiasis | KEGG | 0.000227 |
| map04725 | Cholinergic synapse | KEGG | 0.000316 |
| map04916 | Melanogenesis | KEGG | 0.000363 |
| map04730 | Long-term depression | KEGG | 0.000526 |
| map00230 | Purine metabolism | KEGG | 0.000566 |

**Table S13. The top 20 enrichment of 307 genes introgressed from wild yak to domestic yak.**

| ID | Term | Class | Pvalue |
| --- | --- | --- | --- |
| GO:0042995 | cell projection | CC | 3.84E-05 |
| GO:0005509 | calcium ion binding | MF | 0.000133 |
| GO:0048514 | blood vessel morphogenesis | BP | 0.000235 |
| GO:0030425 | dendrite | CC | 0.000256 |
| GO:0006200 | ATP catabolic process | BP | 0.000301 |
| GO:0009158 | ribonucleoside monophosphate catabolic process | BP | 0.000412 |
| GO:0009169 | purine ribonucleoside monophosphate catabolic process | BP | 0.000412 |
| GO:0009128 | purine nucleoside monophosphate catabolic process | BP | 0.000434 |
| GO:0009125 | nucleoside monophosphate catabolic process | BP | 0.000456 |
| GO:0009653 | anatomical structure morphogenesis | BP | 0.000485 |
| GO:0001948 | glycoprotein binding | MF | 0.000697 |
| GO:0005005 | transmembrane-ephrin receptor activity | MF | 0.000707 |
| GO:0001568 | blood vessel development | BP | 0.000725 |
| GO:0043005 | neuron projection | CC | 0.000788 |
| GO:0043167 | ion binding | MF | 0.000807 |
| GO:0001944 | vasculature development | BP | 0.001003 |
| GO:0097458 | neuron part | CC | 0.001233 |
| GO:0006470 | protein dephosphorylation | BP | 0.001243 |
| GO:0051049 | regulation of transport | BP | 0.00132 |
| GO:0030375 | thyroid hormone receptor coactivator activity | MF | 0.0014 |
| map04520 | Adherens junction | KEGG | 0.000736 |
| map00512 | Mucin type O-glycan biosynthesis | KEGG | 0.001803 |
| map05412 | Arrhythmogenic right ventricular cardiomyopathy (ARVC) | KEGG | 0.004874 |
| map04530 | Tight junction | KEGG | 0.011601 |
| map04974 | Protein digestion and absorption | KEGG | 0.013593 |
| map04120 | Ubiquitin mediated proteolysis | KEGG | 0.01997 |
| map04510 | Focal adhesion | KEGG | 0.020302 |
| map02010 | ABC transporters | KEGG | 0.031449 |
| map00730 | Thiamine metabolism | KEGG | 0.060427 |
| map00532 | Glycosaminoglycan biosynthesis - chondroitin sulfate / dermatan sulfate | KEGG | 0.069212 |
| map04360 | Axon guidance | KEGG | 0.072506 |
| map00514 | Other types of O-glycan biosynthesis | KEGG | 0.094278 |
| map04514 | Cell adhesion molecules (CAMs) | KEGG | 0.095863 |
| map00534 | Glycosaminoglycan biosynthesis - heparan sulfate / heparin | KEGG | 0.096788 |
| map04330 | Notch signaling pathway | KEGG | 0.110127 |
| map00460 | Cyanoamino acid metabolism | KEGG | 0.117214 |
| map04972 | Pancreatic secretion | KEGG | 0.119804 |
| map03440 | Homologous recombination | KEGG | 0.121708 |
| map04723 | Retrograde endocannabinoid signaling | KEGG | 0.134311 |
| map04745 | Phototransduction | KEGG | 0.137406 |
